# High-throughput transcriptome sequencing and preliminary functional analysis in four Neotropical tree species

**DOI:** 10.1186/1471-2164-15-238

**Published:** 2014-03-27

**Authors:** Louise Brousseau, Alexandra Tinaut, Caroline Duret, Tiange Lang, Pauline Garnier-Gere, Ivan Scotti

**Affiliations:** INRA, UMR 0745 EcoFoG, Campus agronomique BP 709, F-97387 Cedex Kragujevac, France; INRA, UMR 1137 EEF, allée de l’Arboretum, 54280 Champenoux, French Guiana; University of French West Indies and French Guiana, UMR EcoFoG, Campus agronomique BP 709, F-97387 KOUROU, Cedex Kragujevac, French Guiana; Key Laboratory of Tropical Forest Ecology, Xishuangbanna Tropical Botanical Garden, Chinese Academy of Sciences, Mengla, Yunnan 666303 China; INRA, UMR 1202 BIOGECO, F-33610 Cestas, France; BIOGECO, UMR 1202, University of Bordeaux, F-33400 Talence, France

**Keywords:** 454-Pyrosequencing, Tropical rainforest tree species, Polymorphism discovery

## Abstract

**Background:**

The Amazonian rainforest is predicted to suffer from ongoing environmental changes. Despite the need to evaluate the impact of such changes on tree genetic diversity, we almost entirely lack genomic resources.

**Results:**

In this study, we analysed the transcriptome of four tropical tree species (*Carapa guianensis*, *Eperua falcata*, *Symphonia globulifera* and *Virola michelii*) with contrasting ecological features, belonging to four widespread botanical families (respectively Meliaceae, Fabaceae, Clusiaceae and Myristicaceae). We sequenced cDNA libraries from three organs (leaves, stems, and roots) using 454 pyrosequencing. We have developed an R and bioperl-based bioinformatic procedure for *de novo* assembly, gene functional annotation and marker discovery. Mismatch identification takes into account single-base quality values as well as the likelihood of false variants as a function of contig depth and number of sequenced chromosomes. Between 17103 (for *Symphonia globulifera*) and 23390 (for *Eperua falcata*) contigs were assembled. Organs varied in the numbers of unigenes they apparently express, with higher number in roots. Patterns of gene expression were similar across species, with metabolism of aromatic compounds standing out as an overrepresented gene function. Transcripts corresponding to several gene functions were found to be over- or underrepresented in each organ. We identified between 4434 (for *Symphonia globulifera*) and 9076 (for *Virola surinamensis*) well-supported mismatches. The resulting overall mismatch density was comprised between 0.89 (*S. globulifera*) and 1.05 (*V. surinamensi*s) mismatches/100 bp in variation-containing contigs.

**Conclusion:**

The relative representation of gene functions in the four transcriptomes suggests that secondary metabolism may be particularly important in tropical trees. The differential representation of transcripts among tissues suggests differential gene expression, which opens the way to functional studies in these non-model, ecologically important species. We found substantial amounts of mismatches in the four species. These newly identified putative variants are a first step towards acquiring much needed genomic resources for tropical tree species.

**Electronic supplementary material:**

The online version of this article (doi:10.1186/1471-2164-15-238) contains supplementary material, which is available to authorized users.

## Background

The Amazonian rainforest hosts one of the greatest pools of terrestrial biodiversity, including very large tree species diversity [1–3]. In forest genetics, most efforts so far have focused on temperate and boreal tree species. While ongoing anthropogenic climate change is suspected to deeply affect the stability of Neotropical rainforests [4], tropical tree species genetic resources and adaptive potential are still poorly known [5], despite the availability of sequence data for several species [6–8]. Identification of polymorphisms and robust estimates of tropical tree species’ standing genetic diversity are thus needed to evaluate the vulnerability to environmental changes of populations and their ability to endure them [9, 10].

A thorough assessment of tropical tree species’ genetic diversity requires large amounts of genomic data and informative molecular markers [11, 12]. Single-nucleotide polymorphisms (SNPs) have become the most popular genome-wide genetic markers [13, 14] and are increasingly used to characterize potentially adaptive genetic variation (e.g. [15–17]).

High-throughput sequencing and genotyping methods have paved the way to genomic studies in non-model species [14, 18, 19], by permitting cost-effective sequencing and the generation of very large genetic data collections. Thus, NGS provides a valuable tool to describe genome properties and variation in non-model species [14, 20]. While assembling whole genomes without a reference sequence can be very complex and in the best cases incomplete, transcriptome sequencing constitutes an efficient alternative in information-poor organisms [21]. Transcriptomes also include a large number of loci with known or predictable functions [22, 23] and have been applied to comparative genomics [24], marker discovery [25], and population genomic studies [26].

An array of next-generation sequencing strategies, varying in read length range and absolute throughput [27] can be used to sequence transcriptomes. The Roche 454-pyrosequencing technology, in spite of being the oldest among these, is the one producing on average the longest reads [23, 28, 29], which makes *de novo* assembly easier in non-model species without prior genomic resources [25, 30, 31] and allows preliminary screening of DNA variation [32] and transcriptome analysis (gene expression profiling by mRNA identification and quantification; [33]).

In this study we describe the transcriptomes of four widespread Neotropical tree genera chosen to represent different botanical families, ecological properties and patterns of local and range distribution (see Methods).

The objectives of the present study are (i) to describe the transcriptomes of these four tropical genera, (ii) to compare expression profiles among species and organs (leaves, stems and roots), and (iii) to provide an initial catalogue of well-supported mismatches, as candidates for validation as SNPs.

## Methods

### Study species and sampling

The four species studied (*Symphonia globulifera* L. f. (Clusiaceae); *Virola surinamensis* (Rol. ex Rottb.) Warb.; *Carapa guianensis* Aubl. (Meliaceae); *Eperua falcata* Aubl. (Fabaceae)) are characterized by contrasting ecological requirements and seed dispersal strategies (Table 1) [34–43]. For each species, we collected about ten seeds from three different sampling sites: Paracou (5°16’20”N; 52°55’32”E) for *E. falcata* and *V. surinamensis*, Matiti (5°3’30”N; −52°36’17”E) for *S. globulifera*, and Rorota (4°51’32”N; −52°21’37”E) for *C. guianensis*. The study complies with the Convention on Biological Diversity. The collection was performed according to local and national legislation on the protection of biodiversity in sampling sites without any special protection status; all sampling permissions were acquired within the frame of the PO-FEDER “ENERGIRAVI” program, granted by the European Union and the Regional government, and by owners of sampling sites (CIRAD for Paracou, Lycée Agricole Matiti for Matiti, ONF for Rorota). The study species are not listed as Endangered by the CITES convention. All the data obtained in this study were shared with the local Regional authorities in compliance with benefit-sharing principles. Seeds germinated and seedlings developed in a greenhouse during twelve months under non-limiting light and water conditions as described in Baraloto et al. [44]. Two vigorous seedlings of each species were selected for transcriptome analyses. Plant material was sampled from three organs: leaves, stems and roots.

**Table 1:** **Species description: distribution range, ecological properties relative to light (successional status) and soil, spatial population structure and seed dispersal properties**

Species name	Range	Ecology - light	Ecology-soil	Spatial population structure	Seed dispersal
*Carapa guianensis*	Neotropics [33]	Light-responsive [34]	Indifferent [34]	Non-aggregated [35]	Gravity, rodents [36]
*Eperua falcata*	Guiana shield [37]	Shade tolerant [34]	Mostly seasonally flooded [34]	Aggregated [38]	Gravity [37]
*Symphonia globulifera*	Neotropics, paleotropics [39]	Shade tolerant [34]	Seasonally flooded [34]	Non-aggregated [35]	Gravity, vertebrates [40]
*Virola surinamensis*	Neotropics [41]	Light-responsive [34]	Seasonally flooded [34]	Non-aggregated [35]	Large vertebrates [42, 43]

### cDNA library preparation and sequencing

Total RNA from each fresh sample was extracted using a CTAB protocol as described by Le Provost *et al.*, [45] (with minor modifications for a subset of the samples). mRNAs were converted to double stranded cDNA using either SMARTer PCR cDNA Synthesis Kit (Clontech) or Mint cDNA synthesis kit (Evrogen) according to the manufacturer’s instructions.

For each species, cDNA libraries from the different organs (leaves, stems and roots) were identified by a specific molecular identifier (MID) tag. Samples from the same organ of different conspecific individuals were pooled for sequencing (MID1 = leaves, MID2 = stems, MID3 = roots). Libraries of the different species were sequenced separately (one run per species) according to a standard Roche-454 protocol [46]. The raw data were submitted to the European Nucleotide Archive (ENA) database (study number: PRJEB3286; http://www.ebi.ac.uk/ena/) and given the accession numbers ERS177107 through ERS177110.

### Assembly and functional annotation

**Figure 1:**
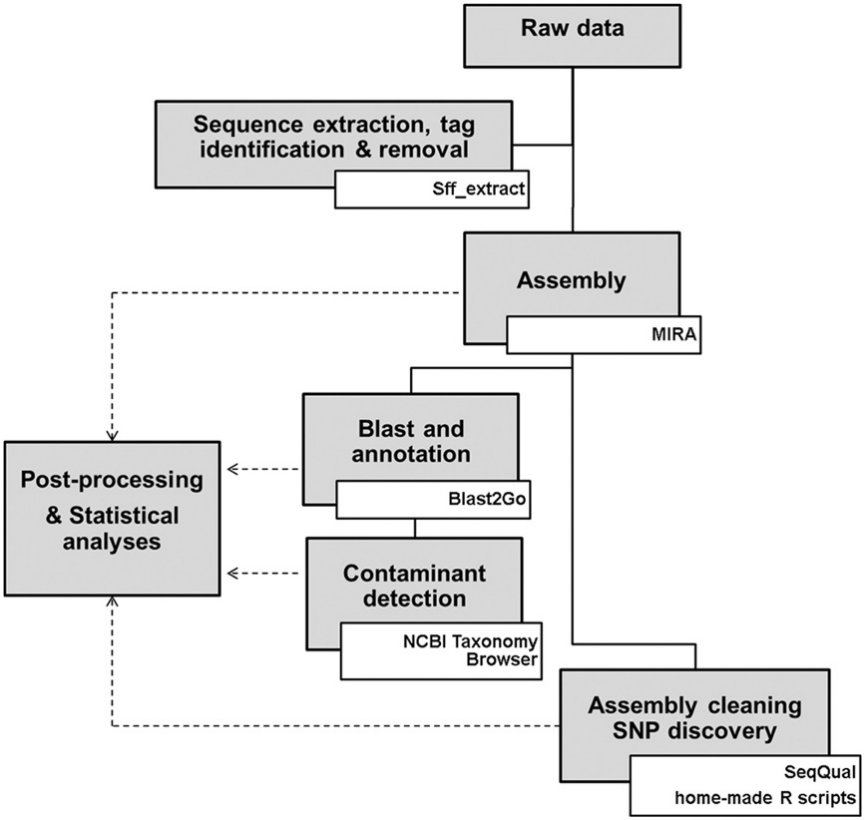
**Bioinformatics flowchart.**

The bioinformatic flowchart includes the following steps (Figure 1): for each species, .sff files were extracted into .fasta, .fasta.qual and .fastq files using the ‘.sff extract’ script available at http://bioinf.comav.upv.es/sff_extract/. The extraction was made both with and without clipping of read ends. Adaptor and MID sequences were identified in .fasta files (with unclipped ends) by searching exact motifs of MID1, MID2 and MID3 in the first twenty bases of each read. The distribution of clipped-end raw read sizes for all species is shown in Additional file 1: Figure S1.

Clipped-ends reads were *de novo* assembled into contigs using MIRA v.3.4.0. The software is rather flexible, has a large range of parameter choices [47] and it has been used efficiently for transcriptome assemblies [48]. We applied the “accurate” mode (with ‘job’ arguments: ‘de novo, est, accurate’) to limit the assembly of paralogous genes. Singletons (i.e. unassembled reads) were discarded for all subsequent steps.

Because different numbers of reads were obtained from different organs, comparisons in the number of contigs (unigenes) among organs may suffer from ascertainment bias, with libraries containing fewer reads displaying fewer contigs due to more limited sampling. To test for this effect we have applied the RaBoT method [49], which compares observed values of a given statistic (here, number of contigs) in a smaller sample (the ‘empirical’ value) with the value obtained from repeated sub-samples of the same size, drawn from a larger sample (the ‘bootstrapped’ values). The statistic in the larger sample is thus evaluated in the same conditions as in the smaller one, which allows an unbiased comparison and their difference to be tested statistically. RaBoT was applied with *N* = 100 sub-samples. Because the sub-samples were not independent, only the non-parametric test and *P-*value (i.e. the fraction of the distribution of ‘bootstrapped’ values that is above the ‘empirical’ value) are reported.

Assembled contig consensus sequences were submitted to Blast2Go (B2G) analysis (http://www.blast2go.de/b2ghome), which permits large-scale blasting, mapping and annotation of novel sequence data particularly in non-model species [50]. BlastX search was performed on species assemblies against the NCBI non-redundant protein database (with BlastX minimum e-value of 10^−3^, Number of Blast Hits = 20). We realized a semi-automated search for contaminants by verifying the organism identity of each blast hit as follows: NCBI Taxonomy CommonTree Browser (http://www.ncbi.nlm.nih.gov/Taxonomy/CommonTree/wwwcmt.cgi) was searched with a non-redundant list of species extracted from B2G. Contigs for which at least one of the ten hits with the lowest e-values (< 10^−25^) identified a sequence from a genus belonging to the "green plant" node of the generated tree were further considered as non-contaminants; contigs with no hits to any “green plant” genus were treated as contaminants and excluded. Contigs were then assigned to the minimum e-value informative functional annotations from plant species hits, provided that their e-value was smaller than 10^−25^.

The Gene Ontology annotation analysis (with e-value hit filter = 10^−6^, Annotation cutoff = 55, GO weight = 5, Hsp-hit coverage cutoff = 0) allowed the matching of each contig with Molecular functions, Cellular Components and Biological processes under the plant GOslim option. Annotation analyses were performed in all cases at levels 3 and 4, that is, with GO terms being three or four nodes away from the root of the GO term trees [51, 52]. These levels were chosen because they group genes according to processes at intermediate levels of biological integration, which can be readily interpreted in terms of implication in cell-, organ- and organism-level developmental and physiological functions [53]. Across-species sharing of *Level 3* GO terms was inspected*.* Moreover, considering that a contig’s number of reads is a rough estimator of the level of expression of the corresponding gene [28, 54, 55], we used the number of reads belonging to contigs associated to *level 4* GO terms to identify processes with organ-specific variations in expression levels. To identify those processes, we used a permutation analysis as follows:

(i) The contigs associated to each *level 4* GO term were identified, and the number *R*_ci_ of reads obtained for each contig i from each organ was recorded. The following steps were executed separately for each organ;
(ii) the *observed* average number of reads across all contigs associated to a given Biological Process  was computed; this statistic was considered as an estimator of the average expression level of all genes involved in that Biological Process (contigs with zero counts were excluded);
(iii) then, the values *R*_ci_ of reads per contig (within each organ) were permuted over all contigs 1000 times. At each permutation, the average read count of all contigs associated to a given biological process  was recorded again, and the difference between empirical (observed)  and  was recorded.
(iv) the distribution of thses differences indicates how close to to average is the expression of genes belonging to a given GO term; i.e., for a Biological Process whose genes exhibit an average level of expression, the distribution of mean differences obtained from permutations overlaps zero; biological processes whose genes have expression levels above average have a distribution of permuted differences above zero, and vice versa for biological processes with genes showing less than average expression levels.
(v) if, for a given biological process, the *observed* average read count per contig was larger than 95% of the average values obtained by permutation, then the group of genes associated to that biological process was considered as over-expressed, and consequently the biological process was considered functionally important for that organ.

Because a contig may be associated to different biological processes, steps (ii)-(v) above were performed for each biological process separately. Because all permutation tests were performed within organs, this analysis is not prone to biases in the number of reads per organ (see above and Results and Discussion). Comparisons among organs for variations in expression among processes were done qualitatively.

### Mismatch identification

Assemblies were post-processed using both bioperl scripts from the SeqQual pipeline (Lang et al. in preparation) (Additional file 2), and home-made R scripts (Additional file 3) that followed various steps of filtering the data by integrating a number of quality criteria (and Additional file 4: Table S1 for a description of programs used). The different steps of the procedure used were as follows:

#### Splitting .ace assembly files and linking to quality

Assembled contig sequence files were extracted from the .ace files given by MIRA and linked to their original base quality scores contained in the .fasta.qual files.

#### Assembly cleaning

Nucleotide differences were screened in assembled contigs and particular bases were masked according to several criterions:

- being a singleton
- being a variant with a frequency lower than 0.1 (see also *4.3* below)
- having a quality score lower than 20 for polymorphic sites.

Following this ‘masking step’, a ‘cleaning step’ removed all positions (i.e. corresponding to one base) of the assembled contigs that contained only indels and masked bases. This last step is particularly relevant for 454 data where false insertions due to homopolymers were very common and drastically affect contig consensus, hampering further re-sequencing and SNP design for genotyping. Consensus (using IUPAC codes) were edited from cleaned assembled data and used both for estimating the total transcriptome length obtained and for identifying well supported mismatches.

#### Computing mismatch statistics and post-filtering

All mismatches contained in the cleaned assemblies were used to build a summary statistics table (number of occurrences and frequency of the different variants, depth, mean quality, minor allele frequency (maf)). This table was used to identify the highest-quality mismatches *a posteriori* (without affecting assembly and consensus). In particular, we chose to avoid:

- mismatches adjacent to each other, because they are likely to be assembly artefacts [56, 57].
- mismatches with lower-than-expected frequencies based on the number of gametes sequenced. With two genotypes, four different gametes were sequenced with the probability of having a variant being 0.25 at minimum. The following rationale can be applied to any number of gametes 2N. The probability of observing a particular number of times (or fewer) the minority variant (with expected frequency in the sequence pool, p=1/2N) follows a binomial distribution. The probability of observing the variant exactly *t* times out of *x* reads is computed as  and the probability of observing it *t* times or fewer is given by . All polymorphisms that were present in a configuration (e.g. 3 variants among 29 reads) with a cumulative probability *P* < 0.05 were considered as false positives and were discarded. This led to the exclusion of additional variants with frequencies between 0.1 and 0.15 but with probability below 5%.
- mismatches having a depth lower than 8X, which can be considered as a stringent criteria, given the 20 quality score for each base, a minimum SNP frequency of 2/8= 0.25 here (since singletons have been previously excluded), and the fact that this configuration has a probability of 0.31 based on the binomial distribution rationale, which is well above the 5% threshold chosen before.

Following the filtering steps described above, mismatches were counted and their density per base was computed as the total number of putative variants (including those at contig ends that passed the quality and singleton filters) divided by the total number of bases where the depth was at least 8 reads. Numbers of transitions, transversions, and deletions were also reported.

## Results and discussion

### Assembly

Sequence data were obtained from all tissues and species except *S. globulifera*, for which root cDNA sequencing failed. Between 167140 and 248145 reads were obtained per species; the distribution of clippend-end read length distributions is shown in Additional file 1: Figure S1. More reads were associated with roots than with stems or leaves (Table 2). This is in agreement with the higher levels of gene expression which were found in the roots compared to other organs in model species such as *Arabidopsis thaliana*[58]. Alternatively, this may be due to technical artefacts such as a more efficient RNA extraction and/or cDNA amplification from roots than from other organs, and a lower RNA extraction yield in leaves due to high concentrations of secondary metabolites. Nevertheless, all RNA samples were equally stable as no sign of degradation was detected after a two-hour incubation at 37 °C (data not shown). Also technical descriptors of the experiment such as RNA A_260_/A_280_ ratio, total amount of RNA used and total cDNA yield did not influence the number of reads, as shown by the non-significant *P*-values associated to each factor in a Generalised Linear Model (GLM; see Additional file 5: Table S2).

**Table 2:** **Partitioning of reads among different organs (leaves, stems, roots) in each species cDNA library (** ***C. guianensis*** **,** ***E. falcata*** **,** ***S. globulifera*** **and** ***V. surinamensis*** **) with percentages in parenthesis**

Number of reads	***Carapa guianensis***	***Eperua falcata***	***Symphonia globulifera***	***Virola surinamensis***
From leaves [MID1]	63016 [43334 (28%)]	17421 [11417 (9%)]	49894 [32190 (30%)]	31526 [22077 (11%)]
From stems [MID2]	47100 [29720 (20%)]	28362 [18088 (14%)]	110373 [66874 (66%)]	41435 [28284 (14%)]
From roots [MID3]	132030 [77052 (50%)]	175551 [100909 (76%)]	7 [2 (0%)]	141948 [89918 (72%)]
Without tag	5999 [3435 (2%)]	3260 [1799 (1%)]	6866 [4367 (4%)]	4314 [2691 (2%)]

Numbers of assembled reads are shown in brackets.

**Figure 2:**
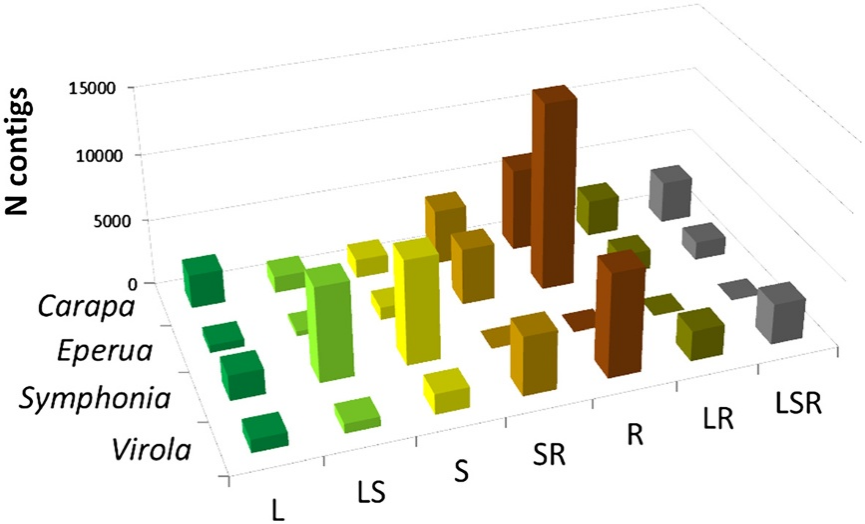
**Number of contigs associated with each organ (leaves, stems, roots) (Note: sequencing from** ***S. globulifera*** **roots failed).** *Carapa* = *Carapa guianensis*; *Eperua* = *Eperua falcata*; *Symphonia* = *Symphonia globulifera*; *Virola* = *Virola surinamensis*. L, S and R indicate contigs specific to Leaf, Stem and Root, respectively; combinations of symbols correspond to contigs occurring in multiple organs.

Between 103433 (*S. globulifera*) and153551 (*C. guianensis*) reads were successfully assembled into contigs and between 17103 and 23390 contigs were obtained, depending on the species (Table 3). These figures are close to the average number of contigs commonly obtained in similar studies [51, 59, 60] and suggest reasonable transcriptome coverage from the data if we assume that the number of contigs slightly overestimates (i.e. multiple contigs may come from the same transcript) the species’ unigene set. However, we expect genes with low expression levels to be missing from our catalogue, as the absolute numbers of reads obtained here prevents assembly of under-represented transcripts. Average contig length varied between 414 bp (*E. falcata*) and 523 bp (*C. guianensis*), and N_50_ values were just above average contig length for all species (Table 3 and Additional file 6: Figure S2); clearly, coverage of individual transcripts and representation of the transcriptome are only partial, and require extension with new sequencing actions, based on higher-throughput methods. The distribution of reads over contigs was quite even, but the coverage was low, with an average between 6 and 7 reads per contig and around 90% of the contigs with 10 reads or fewer (Table 3, Additional file 7: Figure S3). The number of contigs associated to each organ (i.e., the number of contigs including reads from a particular organ or combination of organs) varied widely (Figure 2); to check whether this was an artefact of the absolute number of reads obtained from each organ (Table 2), numbers of contigs obtained from each organ were submitted to RaBoT analyses. In all pairwise comparisons between organs, the number of contigs obtained from the organ with the larger number of reads remained larger after rarefaction (*P*-value = 1 in all comparisons, with the exception of the stem/leaves pair in *C. guianensis*, which had *P* = 0.010, indicating that the difference in number of contigs between these two samples is probably artefactual). Therefore, the larger number of contigs observed in organs with larger number of reads cannot generally be explained entirely by sampling bias. A large number of contigs was solely associated to roots for the three species (Figure 2), particularly in *E. falcata* (61% of contigs from roots only, compared to 29% and 37% for *C. guianensis* and *V. surinamensis*). In contrast, contigs exclusive to stems and leaves were in much lower proportions in the three species with root data, varying from 4% to 7% for stems, and 3% to 12% for leaves (Figure 2).

**Table 3:** **Assembly results: number of assembled reads, number of contigs, total transcriptome coverage, average length per contig, and average number of reads per contig**

	***Carapa guianensis***	***Eperua falcata***	***Symphonia globulifera***	***Virola surinamensis***
Number of reads	248145	224554	167140	219223
Number of assembled reads	153551 (61.9%)	132213 (58.9%)	103433 (61.9%)	142970 (65.2%)
Number of contigs	21770	23390	17103	21070
Total length (bp)	11393209	9688583	7743116	9725915
Average length per contig (bp)	523	414	453	462
N_50_	558	441	486	506
Average number of reads per contig	7	6	6	7
Proportion of contigs with 10 reads or fewer	89%	92%	91%	88%

### Functional annotation

Functional annotation based on BlastX and gene ontology analyses allowed classifying contigs into functional groups. A majority of contigs returned a Blast hit result with e-values below 10^−25^ (Additional file 8: Figure S4) for *C. guianensis* (79%), *E. falcata* (69%), *S. globulifera* (74%) and *V. surinamensis* (70%), but only between 48.1% (*E. falcata*) and 64.1% (*C. guianensis*) had functionally informative annotations (Table 4). Less than 3.1% of the characterized contigs were identified as contaminants for any species (1.58%, 3.06%, 2.92% and 0.29% in *C. guianensis*, *E. falcata*, *V. surinamensis* and *S. globulifera* respectively). After removing contaminants, from 12603 (*S. globulifera*) to 16912 unigenes (*C. guianensis*) with e-value < 10^−25^ were retained, that covered 4.75 Mbp (in *S. globulifera*) to 7.75 Mbp (in *C. guianensis*) (Table 4).

**Table 4:** **BlastX statistics per species, performed on consensus sequences obtained from the MIRA assemblies**

	***Carapa guianensis***	***Eperua falcata***	***Symphonia globulifera***	***Virola surinamensis***
No of unigenes that did not return any blast result	4586 (21.1%)	7231 (30.9%)	4463 (26.1%)	6384 (30.3%)
No of blasted unigenes	17184 (78.9%)	16159 (69.1%)	12640 (73.9%)	14686 (69.7%)
[No unigenes after contaminant removal]	[16912]	[15664]	[12603]	[14545]
No of mapped unigenes	15879 (72.9%)	13629 (56.3%)	11639 (68.1%)	13000 (61.7%)
No of annotated unigenes	13962 (64.1%)	11240 (48.1%)	10164 (59.4%)	11073 (52.6%)
Total assembly length without contaminant (bp)	11266552	9501561	7728777	9666680
[Total length of blasted unigenes after removal of contaminant and unigenes with e-values >10^−25^]	[7746737]	[4789056]	[4748202]	[5887279]

**Figure 3:**
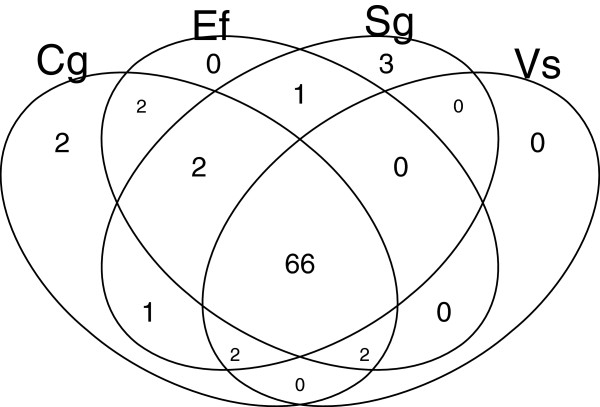
**Sharing of GO terms (level 3) across species.** Only non-contaminant contigs with an e-value lower or equal to 10^−25^ were retained for the analysis. Cg: *Carapa guianensis*; Ef: *Eperua falcata*; Sg: *Symphonia globulifera*; Vs: *Virola surinamensis*.

Gene Ontology analysis provided the annotation of all contigs with significant Blast hits. Additional file 9: Tables S3 and Additional file 10: Table S4 report respectively contig sequences and grouping of contigs by GO term. The more represented GO terms are globally very similar across species for at levels 3 and 4 for Cellular Component, Molecular Function and Biological processes (Additional file 11: Figure S5 and Additional file 12: complementary caption to Figure S5). Interestingly, cyclic and heterocyclic compound-binding (including nucleosides) dominate Molecular functions, with more than 40% of the contigs belonging to such terms (4 and 8, Additional file 11: Figure S5); for comparison, Parchman et al. [61] have found about 20% ‘nucleotide binding’ plus ‘other binding’ in *Pinus contorta*; the excess of functions related to aromatic compounds in tropical trees may suggest a major role of secondary metabolites, as indicated by Cottet et al. [62] for *S. globulifera*. This may be related to the very strong predation pressure exerted by herbivores [63] and pathogens [64] on tropical forest plants. Biological processes (level 4) are dominated by macromolecule metabolism, including again cyclic compound processing, somehow confirming the Molecular function results (‘Response to stress’ ranks fifth in Level 3 Biological processes, with about 4% of the hits, which is compatible with results in [610]). Overall, eighty-one biological process (level 3) were represented for all species (77, 73, 75 and 70 for *C. guianensis*, *E. falcata*, *S. globulifera* and *V. surinamensis*, respectively), of which sixty-six shared by all species, and five represented in only one species (Figure 3); however, the GO terms appearing in only one species were represented by only few contigs (Additional file 13: Table S5). The absolute numbers of contigs belonging to a given GO terms were highly correlated among species (r>0.99 for all pairs; Additional file 14: Figure S6). Given the differences noted above with a conifer species, this strong convergence among tropical species belonging to different families may reflect specific patterns to tree species that undergo the same environmental conditions rather than general patterns in plants.

**Figure 4:**
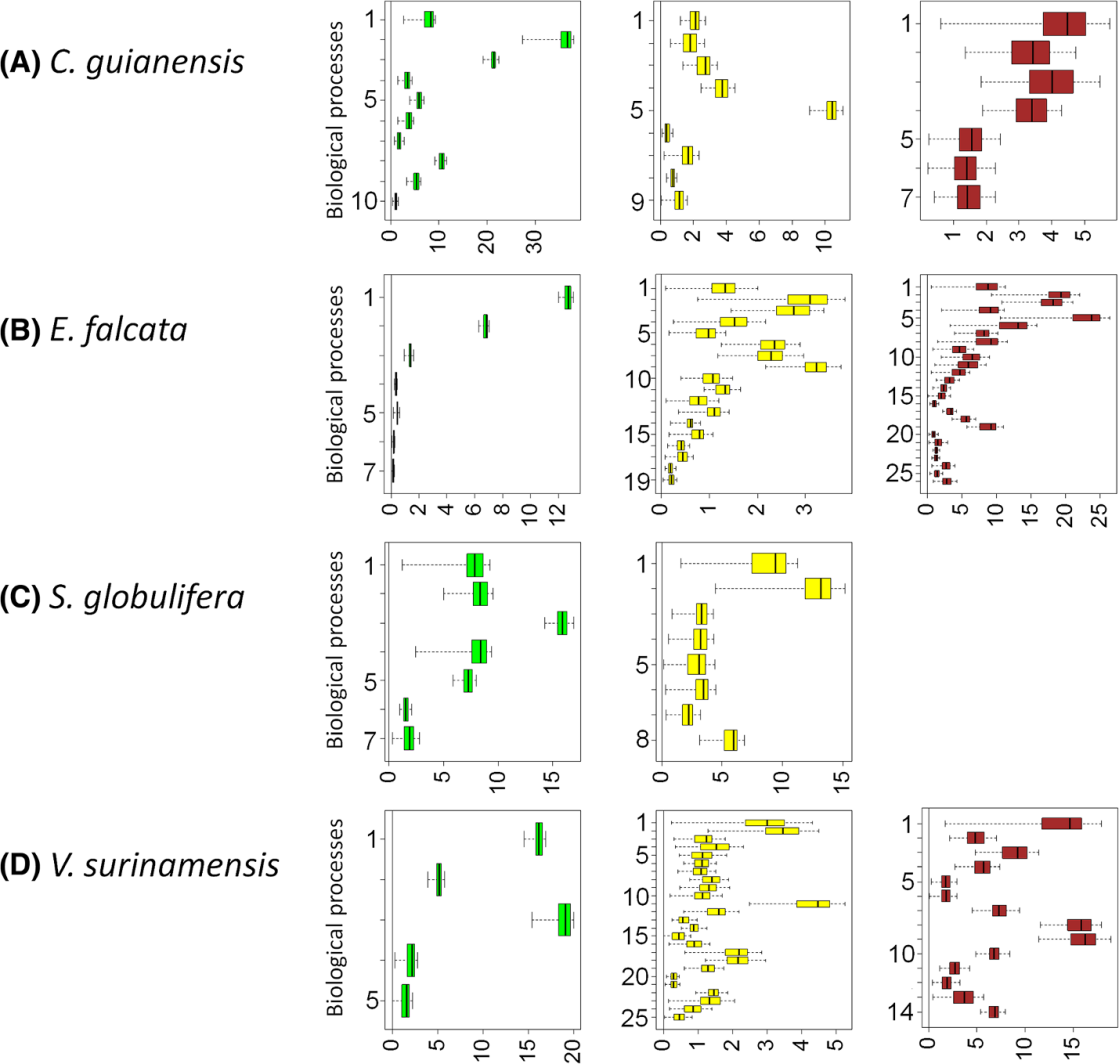
**Box-plot of permuted values of differences between observed and randomised**  **for individual GO terms in each organ/species.** Only biological processes showing a positive difference (i.e. having a bootstrap interval that does not overlap zero, indicating higher expression levels than average) are shown. For detailed names of the biological processes shown, see Additional file 15. **(A)** *C. guianensis*; **(B)** *E. falcata*; **(C)** *S. globulifera*; **(D)** *V. surinamensis* (Note: sequencing from *S. globulifera* roots failed).

Permutation analyses allowed us to identify biological processes (level 4) showing a significantly higher occurrence of contigs for a given organ, that could be interpreted as a higher expression of genes belonging to that process in that organ (Figure 4 and Additional file 15: complementary caption to Figure 4). In leaves, between five (*V. surinamensis*) and ten (*C.guianensis*) biological processes stood out (Figure 4 left column), and eight of them were identified in more than one species. Not surprisingly, biological processes related to photosynthesis and carbon cycle in leaves appear in this group (‘carbohydrate metabolic process’, ‘carbon fixation’, ‘generation of precursor metabolites and energy’, ‘nitrogen cycle metabolic process’, ‘organic substance biosynthetic process’, ‘oxidation reduction process’, ‘photosynthesis’, ‘response to radiation’).

In stems, we detected between eight (*S. globulifera*) and twenty-five (*V. surinamensis*) biological processes (Figure 4 middle column) that had significantly higher-than-average expression levels, fifteen of them being shared among different species. At least a subset of these processes (‘cellular biosynthetic process’, ‘cellular component movement’,‘organic substance biosynthetic process’, ‘organic substance catabolic process’,‘secondary metabolic process’) are potentially related to cell differentiation events that occur during wood formation.

In roots, between seven (*C. guianensis*) and twenty-six (*E. falcata*) biological processes appeared as particularly over-expressed, eleven being shared by different species. They reflect two main functions of roots: water and nutrient uptake (‘response to inorganic substance’, ‘response to ‘organic substance transmembrane transport’) and response to stresses caused by soil constraints, which fall in two classes: (a) soil water depletion (e.g. ‘response to osmotic stress’) which frequently occurs in tropical rainforests during the dry season; (b) oxidative stresses caused by soil hypoxia, to which the processes ‘reactive oxygen species metabolic process’, ‘response to oxidative stress’, and ‘response to oxygen containing compound’ are related; flooding-induced hypoxia is particularly frequent in water-logged bottomlands.

rRNA intron-encoded homing endonucleases were very abundant in the *E. falcata* assembly (581 unigenes against 43, 39 and 17 unigenes in *C. guianensis*, *S. globulifera* and *V. surniamensis* respectively). In *E. falcata*, these unigenes comprised between two and 920 reads with a mean of 15.3 (s.d.=69.77). Homing endonucleases from group I introns are self-splicing genetic elements or parasitic genes mostly found in organellar genomes [64–66]. Among contigs that showed BLASTX hits with rRNA-intron-encoded homing endonucleases in *E. falcata*, 69 were potentially polymorphic and contained from 1 to 18 mismatches with many haplotypes [67]. High transcription levels of such elements, combined with the high numbers of mutations that they have accumulated, suggests a massive but ancient genome invasion event [67, 68] in the *E. falcata* genome compared to the other three species. The evolutionary implications of transfers of such elements remain poorly understood, because of their ‘super-Mendelian’ inheritance (such elements may be both vertically and horizontally transmitted [69]), and because they have no known function [67].

### Mismatch detection

**Figure 5:**
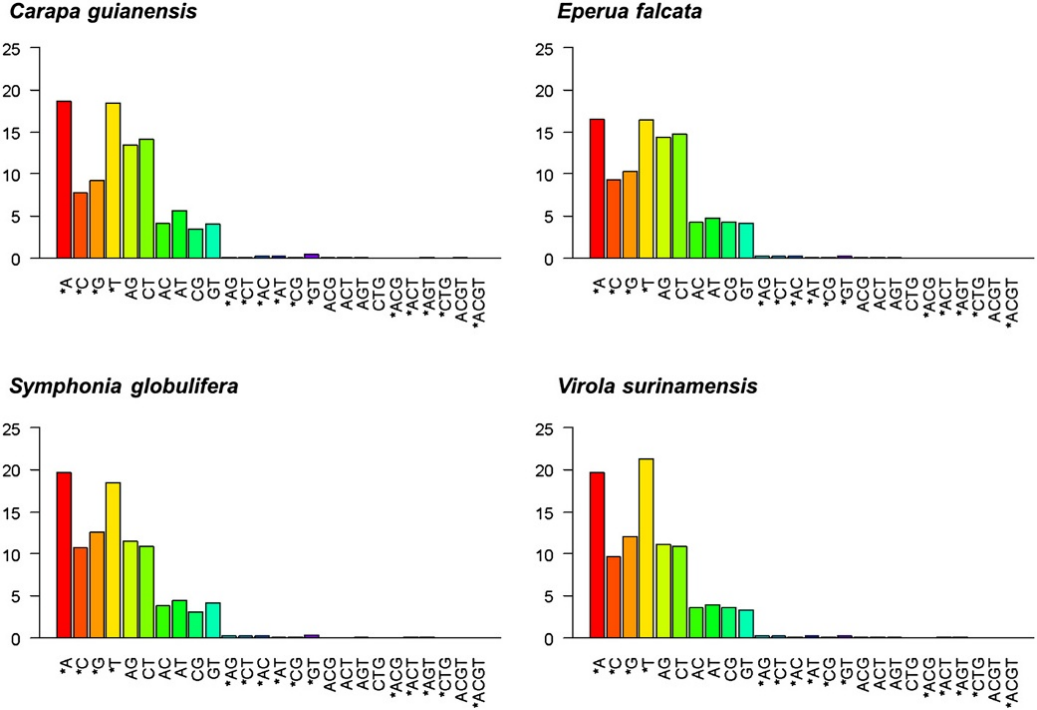
**Mismatches represented based on their allelic pattern.**

It has been shown that relaxed criteria for *in silico* mismatch choice from next-generation sequencing data or previous EST databases leads to high failure rates in subsequent SNP design [70, 71]. We have applied a stringent filtering process based on data quality and a probabilistic argument in order to decrease the frequency of artifactual mismatches. Removal of poor-quality bases in the first steps reduced sequencing depth at mismatch positions from ~20-23x to ~16-17 (Additional file 1: Figure S1). Between 4434 (for *S. globulifera*) and 9076 (for *V. surinamensis*) potential variants were retained after all the filtering steps had been applied (Table 5). Between 5.5% (*E. falcata*) and 8.3% (*V. surinamensis*) of contigs contained at least one potential variant (Table 5). The great majority of mismatches (between 95.7% in *C. guianensis* and 99% in *S. globulifera*) were bi-allelic, with a majority of indels (Figure 5). The transition/transversion ratio (Ti/Tv) varied between 1.5 and 1.7, lower than those observed in other exome assemblies [71]. Estimated mismatch density across variable contigs varied between 0.89 per 100 bp (*C. guianensis*) and 1.05 per 100 bp (*V. surinamensis*) (Table 5). These estimates of mismatch density are in the same order of magnitude as SNP density estimates observed in other studies: Parchman *et al.*[72] reported between 0.6 to 1.1 SNPs per 100 bp in *Pinus taeda*, depending on the stringency of their filtering criteria. This may suggest that our mismatch filtering protocol eliminates large amounts of false variants, which would not be validated at the SNP design step. The validation of these mismatches is beyond the scope of this study, and therefore the variants identified here can only be considered as putative, candidate loci for polymorphism. Nevertheless, we advocate for the introduction of stringent criteria for the identification of these putative variants, as more liberal strategies can lead to large numbers of false positives, which lower the efficiency of large-scale SNP screenings.

**Table 5:** **Mismatch identification**

	***Carapa guianensis***	***Eperua falcata***	***Symphonia globulifera***	***Virola surinamensis***
Total length with depth ≥ 8X after assembly cleaning (bases)	956876	603897	499694	862357
**Before post-filtering based on binomial test**				
N mismatches	**10615**	**7084**	**5447**	**10897**
N variant-containing contigs	1716 (7.88%)	1299 (5.55%)	987 (5.77%)	1752 (8.32%)
mismatch density (/100 bp)	1.11	1.17	1.09	1.26
N mismatches with 2 variants	10420 (98.16%)	6968 (98.36%)	5362 (98.44%)	10757 (98.72%)
N transitions	2655	1875	1090	2182
N transversions	1699	1155	779	1474
Ti/Tv	1.56	1.62	1.40	1.48
N indel	6066	3938	3493	7101
N mismatches > 2 variants	195 (1.84%)	116 (1.64%)	85 (1.56%)	140 (1.28%)
**After post-filtering based on binomial test**				
N mismatches	**8646**	**5713**	**4434**	**9076**
N variant-containing contigs	1706 (7.83%)	1283 (5.5%)	979 (5.72%)	1746 (8.29%)
mismatch density (/100 bp)	0.90	0.95	0.89	1.05
N mismatches with 2 variants	8534 (95.70%)	5649 (98.89%)	4388 (98.96%)	8981 (98.95%)
N transitions	2380	1657	989	1989
N transversions	1488	1000	681	1310
Ti/Tv	1.60	1.66	1.45	1.52
N indel	4666	2992	2718	5682
N mismatches > 2 variants	112 (1.13%)	64 (1.12%)	46 (1.04%)	95 (1.05%)

### Candidate transcriptome polymorphism and its usefulness in population genetics studies

Next-generation sequencing, allowing massive *de novo* acquisition of molecular data, provides a range of new potential applications for evolutionary and ecological-genetic studies in non-model species. High-throughput SNP data have indeed shown their potential for inferences about demographic and adaptive processes in natural populations [16, 73–79]; for examples in tree species, see [80, 81]. However, SNP design and validation has often frustratingly low success rates, because candidate variant identification is not stringent enough; in this paper, we have proposed a strategy to filter out false positives based on multiple criteria.

## Conclusion

The genomic resources obtained here will trigger new exciting fields of research on tropical biodiversity. Providing a catalogue of putative functions for genomic regions with a high potential diversity will help identifying useful candidate genes for further resequencing or SNP genotyping [12, 82, 83]. These genes belong to a large range of biological processes, including growth, reproduction, light and nutrient acquisitions, as well as plant response to biotic and abiotic stresses. Focusing on genes potentially involved in adaptive processes in Neotropical forest tree species will permit to test hypotheses about evolutionary processes underlying genome evolution and the build-up of biological diversity in tropical forest ecosystems.

## Availability of supporting data

The raw data were submitted to the ENA database (study number: PRJEB3286) and given the accession numbers ERS177107 through ERS177110.

## Electronic supplementary material

Additional file 1: Figure S1: Clipped-end read length distribution for all species. Representation of mismatch site depths before and after masking procedure. (PDF 7 KB)

Additional file 2: **Methods S1a.** (ZIP 28 KB)

Additional file 3: **Methods S1b.** (ZIP 14 KB)

Additional file 4: Table S1: List and description of the programs used in mismatch discovery. (XLSX 17 KB)

Additional file 5: Table S2: RNA and cDNA quality and quantity data. (a) Species identity: Cg: *Carapa guianensis*; Ef: *Eperua falcata*; Sg: *Symphonia globulifera*; Vs: *Virola surinamensis*. RNA protocol: see Materials and Methods. RNAamount1 and RNAamount2: quantity of RNA (ng) used for cDNA synthesis for each of the two samples used in each species/organ. tot_RNA: sum of RNA amounts from each sample. A260280_1 and A260280_2: absorbance ratios at 260 and 280 nm wavelength for each of the two samples for each species/organ; avg_A260280 and min_ A260280: mean and minimum absorbance ratios calculated from the two samples from each species/organ; cDNA protocol: S = SMARTer cDNA synthesis kit; M = Mint cDNA synthesis kit (see Materials and Methods). cDNAamount1 and cDNAamount2: quantity of cDNA (ng) obtained from each of the two samples for each species/organ; tot_cDNA: cumulative quantity of cDNA obtained; nReads: number of reads obtained. (b) Formula and results of the generalized Linear Model used to test the effect of experimental variables on nReads. (XLSX 16 MB)

Additional file 6: Figure S2: Distribution of contig lengths within each assembly. (TIFF 53 KB)

Additional file 7: Figure S3: Histogram of the number of reads assembled in each contig. The x axis is displayed in log(10) scale. (PDF 5 KB)

Additional file 8: Figure S4: Number of contigs returning a blast result using different e-value thresholds: 10^−5^, 10^−10^, 10^−15^, 10^−20^ and 10^−25^. (TIFF 45 KB)

Additional file 9: Table S3: Contig names and sequences for each species. (XLS 1 MB)

Additional file 10: Table S4: GO terms and the associated contigs for all species. (a) *Carapa guianensis*; (b) *Eperua falcata*; (c) *Symphonia globulifera*; (d) *Virola surinamensis*. (XLS 1 MB)

Additional file 11: Figure S5: Number of contigs (y axis) per GO term per species at levels 3 and 4 for cellular components, molecular functions and biological processes. For analyses where ten or fewer GO terms appear, bars are shown for all GO terms. For analyses where more than ten terms appear, only the bars for the smallest set of terms summing up to 80% of the contigs are shown. Pane 1: Cellular components, level 3; pane 2: Cellular components, level 4; pane 2: Molecular functions, level 3; pane 4: Molecular functions, level 4; pane 5: Biological processes, level 3; pane 6: Biological processes, level 4. Species identity: Cg: *Carapa guianensis*; Ef: *Eperua falcata*; Sg: *Symphonia globulifera*; Vs: *Virola surinamensis*. Identity codes for GO terms: see Additional file 12. (PDF 23 KB)

Additional file 12: **Complementary caption to Figure S5.** Identity codes for GO terms in Additional file 11: Figure S5. (DOCX 13 KB)

Additional file 13: Table S5: Number of contigs belonging to each biological process (GO terms level 3) in each species (the columns sum up to more than the number of assembled contigs because each contig can belong to several GO terms). Cg: *Carapa guianensis*; Ef: *Eperua falcata*; Sg: *Symphonia globulifera*; Vs: *Virola surinamensis*. (XLS 1 MB)

Additional file 14: Figure S6: Species-species pairwise plot of the number of contigs belonging to each biological process (GO terms level 3). (PDF 10 KB)

Additional file 15: **Complementary Caption to Figure** 4 **.** Detailed names of the biological processes shown in Figure 4. (DOCX 12 KB)

## References

[1] Hoorn C, Wesselingh FP, ter Steege H, Bermudez MA, Mora A, Sevink J, Sanmartín I, Sanchez-Meseguer A, Anderson CL, Figueiredo JP, Jaramillo C, Riff D, Negri FR, Hooghiemstra H, Lundberg J, Stadler T, Särkinen T, Antonelli A (2010). Amazonia through time: andean uplift, climate change, landscape evolution, and biodiversity. Science.

[2] Hubbell SP, He F, Condit R, Borda-de-Água L, Kellner J, ter Steege H (2008). How many tree species are there in the Amazon and how many of them will go extinct?. Proc Natl Acad Sci.

[3] Hawkins BA, Rodríguez MÁ, Weller SG (2011). Global angiosperm family richness revisited: linking ecology and evolution to climate. J Biogeogr.

[4] Phillips OL, Aragao LEOC, Lewis SL, Fisher JB, Lloyd J, Lopez-Gonzalez G, Malhi Y, Monteagudo A, Peacock J, Quesada CA, van der Heijden G, Almeida S, Amaral I, Arroyo L, Aymard G, Baker TR, Banki O, Blanc L, Bonal D, Brando P, Chave J, de Oliveira ACA, Cardozo ND, Czimczik CI, Feldpausch TR, Freitas MA, Gloor E, Higuchi N, Jimenez E, Lloyd G (2009). Drought Sensitivity of the Amazon Rainforest. Science.

[5] Savolainen O, Pyhäjärvi T, Knürr T (2007). Gene Flow and Local Adaptation in Trees. Annu Rev Ecol Evol Syst.

[6] Audigeos D, Brousseau L, Traissac S, Scotti-Saintagne C, Scotti I (2013). Molecular divergence in tropical tree populations occupying environmental mosaics. J Evol Biol.

[7] Audigeos D (2010). Relations entre diversité génétique et environnement: quels sont les processus évolutifs mis en jeu ? Cas d’une espèce d’arbre tropical: Eperua falcata Aublet. Ph. D. thesis..

[8] Argout X, Salse J, Aury J-M, Guiltinan MJ, Droc G, Gouzy J, Allegre M, Chaparro C, Legavre T, Maximova SN, Abrouk M, Murat F, Fouet O, Poulain J, Ruiz M, Roguet Y, Rodier-Goud M, Barbosa-Neto JF, Sabot F, Kudrna D, Ammiraju JSS, Schuster SC, Carlson JE, Sallet E, Schiex T, Dievart A, Kramer M, Gelley L, Shi Z, Berard A (2010). The genome of *Theobroma cacao*. Nat Genet.

[9] Scotti I (2010). Adaptive potential in forest tree populations: what is it, and how can we measure it?. Ann For Sci.

[10] Jump AS, Marchant R, Peñuelas J (2008). Environmental change and the option value of genetic diversity. Trends Plant Sci.

[11] Aitken SN, Yeaman S, Holliday JA, Wang T, Curtis-McLane S (2008). Adaptation, migration or extirpation: climate change outcomes for tree populations. Evol Appl.

[12] Stapley J, Reger J, Feulner PGD, Smadja C, Galindo J, Ekblom R, Bennison C, Ball AD, Beckerman AP, Slate J (2010). Adaptation genomics: the next generation. Trends Ecol Evol.

[13] Helyar SJ, Hemmer-Hansen J, Bekkevold D, Taylor MI, Ogden R, Limborg MT, Cariani A, Maes GE, Diopere E, Carvalho GR, Nielsen EE (2011). Application of SNPs for population genetics of nonmodel organisms: new opportunities and challenges. Mol Ecol Resour.

[14] Seeb JE, Carvalho G, Hauser L, Naish K, Roberts S, Seeb LW (2011). Single-nucleotide polymorphism (SNP) discovery and applications of SNP genotyping in nonmodel organisms. Mol Ecol Resour.

[15] Schlötterer C (2002). Towards a molecular characterization of adaptation in local populations. Curr Opin Genet Dev.

[16] Eckert AJ, van Heerwaarden J, Wegrzyn JL, Nelson CD, Ross-Ibarra J, Gonzalez-Martinez SC, Neale DB (2010). Patterns of Population Structure and Environmental Associations to Aridity Across the Range of Loblolly Pine (*Pinus taeda* L., Pinaceae). Genetics.

[17] Eveno E, Collada C, Guevara MA, Leger V, Soto A, Diaz L, Leger P, Gonzalez-Martinez SC, Cervera MT, Plomion C, Garnier-Gere PH (2008). Contrasting Patterns of Selection at Pinus pinaster Ait. Drought Stress Candidate Genes as Revealed by Genetic Differentiation Analyses. Mol Biol Evol.

[18] Allendorf FW, Hohenlohe PA, Luikart G (2010). Genomics and the future of conservation genetics. Nat Rev Genet.

[19] Ellegren H (2008). Sequencing goes 454 and takes large-scale genomics into the wild. Mol Ecol.

[20] Egan AN, Schlueter J, Spooner DM (2012). Applications of next-generation sequencing in plant biology. Am J Bot.

[21] Pop M, Salzberg SL (2008). Bioinformatics challenges of new sequencing technology. Trends Genet.

[22] Bouck AMY, Vision T (2007). The molecular ecologist's guide to expressed sequence tags. Mol Ecol.

[23] Emrich SJ, Barbazuk WB, Li L, Schnable PS (2007). Gene discovery and annotation using LCM-454 transcriptome sequencing. Genome Res.

[24] Vera JC, Wheat CW, Fescemyer HW, Frilander MJ, Crawford DL, Hanski I, Marden JH (2008). Rapid transcriptome characterization for a nonmodel organism using 454 pyrosequencing. Mol Ecol.

[25] Novaes E, Drost D, Farmerie W, Pappas G, Grattapaglia D, Sederoff R, Kirst M (2008). High-throughput gene and SNP discovery in Eucalyptus grandis, an uncharacterized genome. BMC Genomics.

[26] Namroud M-C, Beaulieu J, Juge N, Laroche J, Bousquet J (2008). Scanning the genome for gene single nucleotide polymorphisms involved in adaptive population differentiation in white spruce. Mol Ecol.

[27] Mutz K-O, Heilkenbrinker A, Lönne M, Walter J-G, Stahl F (2013). Transcriptome analysis using next-generation sequencing. Curr Opin Biotechnol.

[28] Weber APM, Weber KL, Carr K, Wilkerson C, Ohlrogge JB (2007). Sampling the Arabidopsis Transcriptome with Massively Parallel Pyrosequencing. Plant physiology.

[29] Wicker T, Schlagenhauf E, Graner A, Close T, Keller B, Stein N (2006). 454 sequencing put to the test using the complex genome of barley. BMC Genomics.

[30] Blanca J, Pascual L, Ziarsolo P, Nuez F, Canizares J (2011). ngs_backbone: a pipeline for read cleaning, mapping and SNP calling using Next Generation Sequence. BMC Genomics.

[31] Margam VM, Coates BS, Bayles DO, Hellmich RL, Agunbiade T, Seufferheld MJ, Sun W, Kroemer JA, Ba MN, Binso-Dabire CL, Baoua I, Ishiyaku MF, Covas FG, Srinivasan R, Armstrong J, Murdock LL, Pittendrigh BR (2011). Transcriptome Sequencing, and Rapid Development and Application of SNP Markers for the Legume Pod Borer *Maruca vitrata* (Lepidoptera: Crambidae). PLOS One.

[32] Barbazuk WB, Emrich SJ, Chen HD, Li L, Schnable PS (2007). SNP discovery via 454 transcriptome sequencing. Plant J.

[33] Morozova O, Marra MA (2008). Applications of next-generation sequencing technologies in functional genomics. Genomics.

[34] Kenfack D (2011). A Synoptic Revision of Carapa (Meliaceae). Harv Pap Bot.

[35] Vincent G, Molino J-F, Marescot L, Barkaoui K, Sabatier D, Freycon V, Roelens J (2011). The relative importance of dispersal limitation and habitat preference in shaping spatial distribution of saplings in a tropical moist forest: a case study along a combination of hydromorphic and canopy disturbance gradients. Ann For Sci.

[36] Degen B, Caron H, Bandou E, Maggia L, Chevallier MH, Leveau A, Kremer A (2001). Fine-scale spatial genetic structure of eight tropical tree species as analysed by RAPDs. Heredity.

[37] Forget P-M, Cuijpers L (2008). Survival and Scatterhoarding of Frugivores-Dispersed Seeds as a Function of Forest Disturbance. Biotropica.

[38] Cowan RS (1975). A monograph of the genus Eperua (Leguminosae: Caesalpinioideae). Smithsonian Contr Bot.

[39] Ter Steege H, Zondervan G, ter Steege H (2000). A preliminary analysis of large-scale forest inventory data of the Guiana Shield. Plant Diversity in Guyana.

[40] Dick Christopher W, Abdulah Salim K, Bermingham E (2003). Molecular systematic analysis reveals cryptic tertiary diversification of a widespread tropical rain forest tree. Am Nat.

[41] Pr A, Hamrick JL, Chavarriaga P, Kochert G (1998). Microsatellite analysis of demographic genetic structure in fragmented populations of the tropical tree *Symphonia globulifera*. Mol Ecol.

[42] Wilson TK, Smith N, Mori SA, Henderson DW, Heald SV (2004). Myristicaceae. Flowering plants of the Neotropics.

[43] Forget PM, Sabatier D (1997). Dynamics of the seedling shadow of a frugivore-dispersed tree species in French Guiana. Journal of tropical ecology.

[44] Baraloto C, Morneau F, Bonal D, Blanc L, Ferry B (2007). Seasonal water stress tolerance and habitat associations within four neotropical tree genera. Ecology.

[45] Le Provost G, Paiva J, Pot D, Brach J, Plomion C (2003). Seasonal variation in transcript accumulation in wood-forming tissues of maritime pine (Pinus pinaster Ait.) with emphasis on a cell wall glycine-rich protein. Planta.

[46] Meyer M, Stenzel U, Hofreiter M (2008). Parallel tagged sequencing on the 454 platform. Nat Protocols.

[47] Chevreux B, Pfisterer T, Drescher B, Driesel AJ, Müller WEG, Wetter T, Suhai S (2004). Using the miraEST Assembler for Reliable and Automated mRNA Transcript Assembly and SNP Detection in Sequenced ESTs. Genome research.

[48] Kumar S, Blaxter M (2010). Comparing de novo assemblers for 454 transcriptome data. BMC Genomics.

[49] Conesa A, Götz S (2008). Blast2GO: A comprehensive suite for functional analysis in plant genomics. Int J Plant Genomics.

[50] Scotti I, Montaigne W, Cseke K, Traissac S (2013). RaBoT: a rarefaction-by-bootstrap method to compare genome-wide levels of genetic diversity. Ann For Sci.

[51] Consortium TGO (2010). The Gene Ontology in 2010: extensions and refinements. Nucleic Acids Res.

[52] Carbon S, Ireland A, Mungall CJ, Shu S, Marshall B, Lewis S, Hub tA (2009). AmiGO: online access to ontology and annotation data. Bioinformatics.

[53] Consortium TGO (2008). The Gene Ontology project in 2008. Nucleic Acids Res.

[54] Torres TT, Metta M, Ottenwälder B, Schlötterer C (2008). Gene expression profiling by massively parallel sequencing. Genome Res.

[55] Frias-Lopez J, Shi Y, Tyson GW, Coleman ML, Schuster SC, Chisholm SW, DeLong EF (2008). Microbial community gene expression in ocean surface waters. Proc Natl Acad Sci.

[56] Craft JA, Gilbert JA, Temperton B, Dempsey KE, Ashelford K, Tiwari B, Hutchinson TH, Chipman JK (2010). Pyrosequencing of *Mytilus galloprovincialis* cDNAs: Tissue-Specific Expression Patterns. PLOS One.

[57] You F, Huo N, Deal K, Gu Y, Luo M-C, McGuire P, Dvorak J, Anderson O (2011). Annotation-based genome-wide SNP discovery in the large and complex Aegilops tauschii genome using next-generation sequencing without a reference genome sequence. BMC Genomics.

[58] Schmid M, Davison TS, Henz SR, Pape UJ, Demar M, Vingron M, Scholkopf B, Weigel D, Lohmann JU (2005). A gene expression map of Arabidopsis thaliana development. Nat Genet.

[59] Sloan DB, Keller SR, Berardi AE, Sanderson BJ, Karpovich JF, Taylor DR (2012). De novo transcriptome assembly and polymorphism detection in the flowering plant Silene vulgaris (Caryophyllaceae). Mol Ecol Resour.

[60] Blanca J, Canizares J, Roig C, Ziarsolo P, Nuez F, Pico B (2011). Transcriptome characterization and high throughput SSRs and SNPs discovery in Cucurbita pepo (Cucurbitaceae). BMC Genomics.

[61] Parchman T, Geist K, Grahnen J, Benkman C, Buerkle CA (2010). Transcriptome sequencing in an ecologically important tree species: assembly, annotation, and marker discovery. BMC Genomics.

[62] Cottet K, Genta-Jouve G, Fromentin Y, Duplais C, Laprévote O, Michel S, Lallemand M-C (2014). Comparative LC-MS-based metabolite profiling of the ancient tropical rainforest tree *Symphonia globulifera*. Phytochemistry.

[63] Lamarre GPA, Baraloto C, Fortunel C, Dávila N, Mesones I, Rios JG, Ríos M, Valderrama E, Pilco MV, Fine PVA (2012). Herbivory, growth rates, and habitat specialization in tropical tree lineages: implications for Amazonian beta-diversity. Ecology.

[64] Bagchi R, Gallery RE, Gripenberg S, Gurr SJ, Narayan L, Addis CE, Freckleton RP, Lewis OT (2014). Pathogens and insect herbivores drive rainforest plant diversity and composition. Nature.

[65] Burt A, Koufopanou V (2004). Homing endonuclease genes: the rise and fall and rise again of a selfish element. Curr Opin Genet Dev.

[66] Cho Y, Qiu Y-L, Kuhlman P, Palmer JD (1998). Explosive invasion of plant mitochondria by a group I intron. Proc Natl Acad Sci.

[67] Yahara K, Fukuyo M, Sasaki A, Kobayashi I (2009). Evolutionary maintenance of selfish homing endonuclease genes in the absence of horizontal transfer. Proc Natl Acad Sci.

[68] Nystedt B, Street NR, Wetterbom A, Zuccolo A, Lin Y-C, Scofield DG, Vezzi F, Delhomme N, Giacomello S, Alexeyenko A, Vicedomini R, Sahlin K, Sherwood E, Elfstrand M, Gramzow L, Holmberg K, Hallman J, Keech O, Klasson L, Koriabine M, Kucukoglu M, Kaller M, Luthman J, Lysholm F, Niittyla T, Olson A, Rilakovic N, Ritland C, Rossello JA, Sena J (2013). The Norway spruce genome sequence and conifer genome evolution. Nature.

[69] Koufopanou V, Goddard MR, Burt A (2002). Adaptation for Horizontal Transfer in a Homing Endonuclease. Mol Biol Evol.

[70] Huse SM, Huber JA, Morrison HG, Sogin ML, Welch DM (2007). Accuracy and quality of massively parallel DNA pyrosequencing. Genome Biol.

[71] DePristo MA (2011). A framework for variation discovery and genotyping using next-generation DNA sequencing data. Nature Genet.

[72] Parchman TL, Gompert Z, Mudge J, Schilkey FD, Benkman CW, Buerkle CA (2012). Genome-wide association genetics of an adaptive trait in lodgepole pine. Mol Ecol.

[73] Nielsen R, Williamson S, Kim Y, Hubisz MJ, Clark AG, Bustamante C (2005). Genomic scans for selective sweeps using SNP data. Genome Res.

[74] Nielsen R, Hubisz MJ, Hellmann I, Torgerson D, Andrés AM, Albrechtsen A, Gutenkunst R, Adams MD, Cargill M, Boyko A, Indap A, Bustamante CD, Clark AG (2009). Darwinian and demographic forces affecting human protein coding genes. Genome Res.

[75] Li H, Stephan W (2006). Inferring the Demographic History and Rate of Adaptive Substitution in Drosophila. PLoS Genet.

[76] Siol M, Wright SI, Barrett SCH (2010). The population genomics of plant adaptation. New Phytol.

[77] Turner TL, Bourne EC, Von Wettberg EJ, Hu TT, Nuzhdin SV (2010). Population resequencing reveals local adaptation of Arabidopsis lyrata to serpentine soils. Nat Genet.

[78] Fournier-Level A, Korte A, Cooper MD, Nordborg M, Schmitt J, Wilczek AM (2011). A Map of Local Adaptation in Arabidopsis thaliana. Science.

[79] Hancock AM, Brachi B, Faure N, Horton MW, Jarymowycz LB, Sperone FG, Toomajian C, Roux F, Bergelson J (2011). Adaptation to Climate Across the Arabidopsis thaliana Genome. Science.

[80] Eckert AJ, Wegrzyn JL, Pande B, Jermstad KD, Lee JM, Liechty JD, Tearse BR, Krutovsky KV, Neale DB (2009). Multilocus Patterns of Nucleotide Diversity and Divergence Reveal Positive Selection at Candidate Genes Related to Cold Hardiness in Coastal Douglas Fir (*Pseudotsuga menziesii* var. *menziesii*). Genetics.

[81] Holliday JA, Suren H, Aitken SN (2012). Divergent selection and heterogeneous migration rates across the range of Sitka spruce (Picea sitchensis). Proc R Soc B-Biol Sci.

[82] Lister R, Gregory BD, Ecker JR (2009). Next is now: new technologies for sequencing of genomes, transcriptomes, and beyond. Curr Opin Plant Biol.

[83] Morozova O, Hirst M, Marra MA (2009). Applications of New Sequencing Technologies for Transcriptome Analysis. Annu Rev Genomics Hum Genet.

