# Supplementary figures and images for: High-throughput transcriptome sequencing and preliminary functional analysis in four Neotropical tree species
Source: BMC Genomics. 2014 Mar 27;15(1):238. doi: 10.1186/1471-2164-15-238 (PMC3986928; doi:10.1186/1471-2164-15-238)

*C. guianensis*

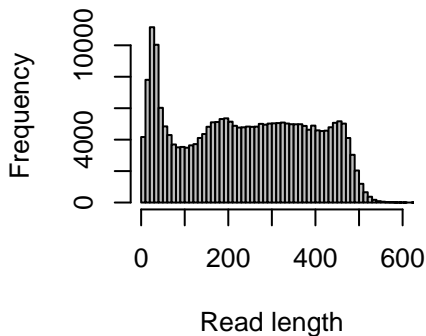

*E. falcata*

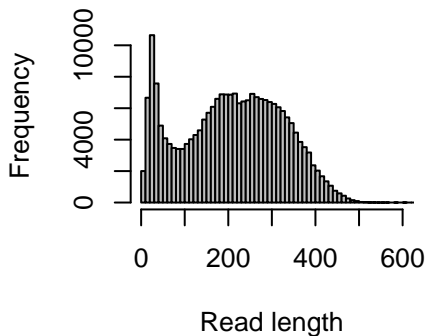

*S. globulifera*

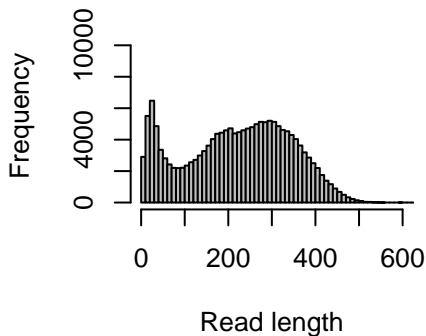

*V. surinamensis*

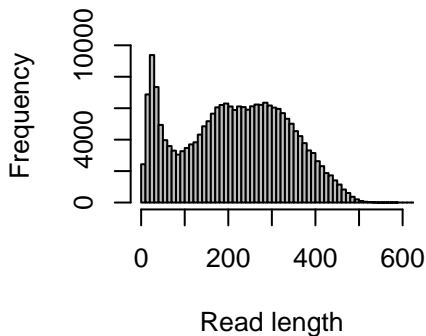

Supplement: Supplementary file 1 — Additional file 1: Figure S1: Clipped-end read length distribution for all species. Representation of mismatch site depths before and after masking procedure. (PDF 7 KB) [file 12864_2014_7044_MOESM1_ESM.pdf]

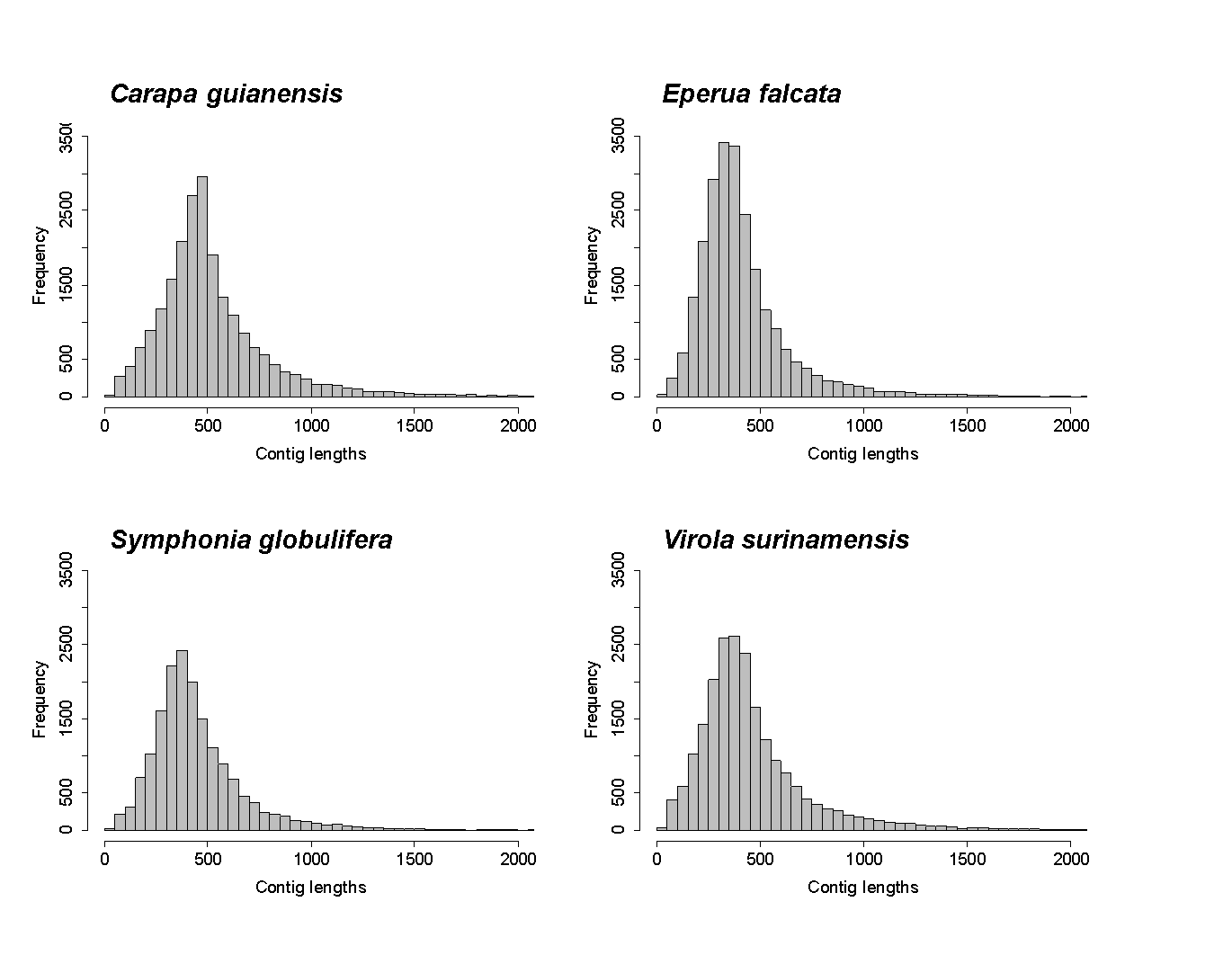

Supplement: Supplementary file 6 — Additional file 6: Figure S2: Distribution of contig lengths within each assembly. (TIFF 53 KB) [file 12864_2014_7044_MOESM6_ESM.tiff]

*C. guianensis*

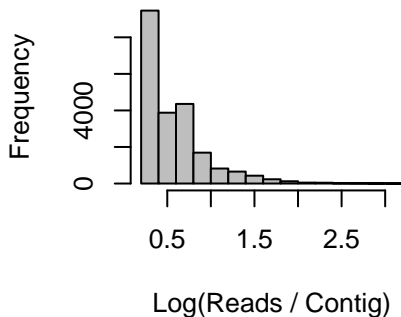

*E. falcata*

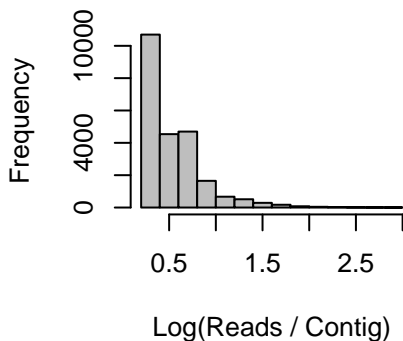

*S. globulifera*

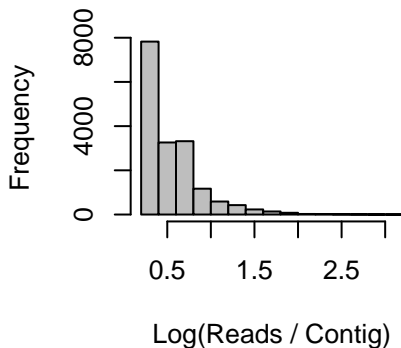

*V. sebifera*

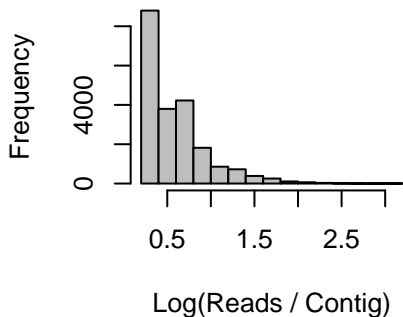

Supplement: Supplementary file 7 — Additional file 7: Figure S3: Histogram of the number of reads assembled in each contig. The x axis is displayed in log(10) scale. (PDF 5 KB) [file 12864_2014_7044_MOESM7_ESM.pdf]

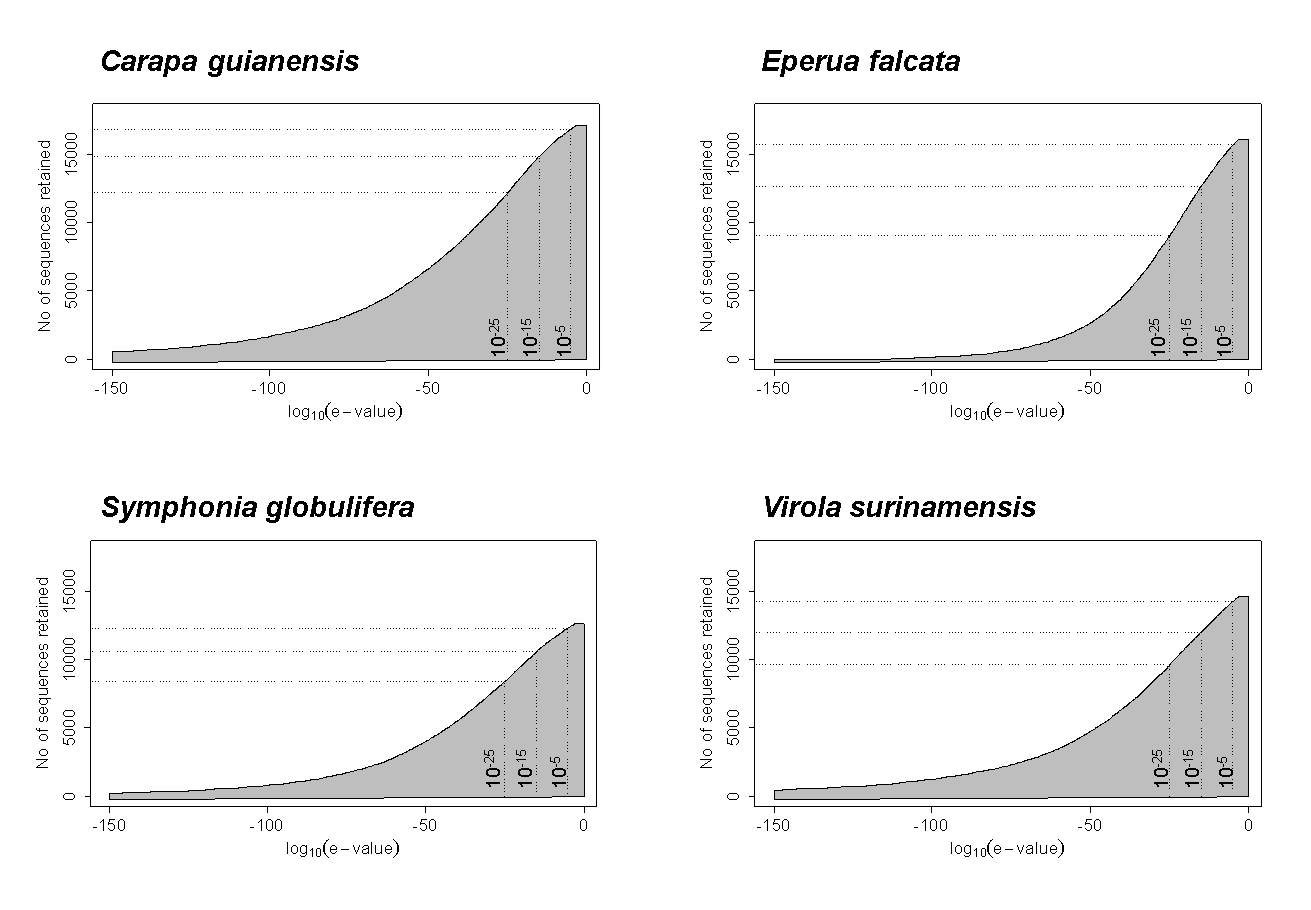

Supplement: Supplementary file 8 — Additional file 8: Figure S4: Number of contigs returning a blast result using different e-value thresholds: 10−5, 10−10, 10−15, 10−20 and 10−25. (TIFF 45 KB) [file 12864_2014_7044_MOESM8_ESM.tiff]

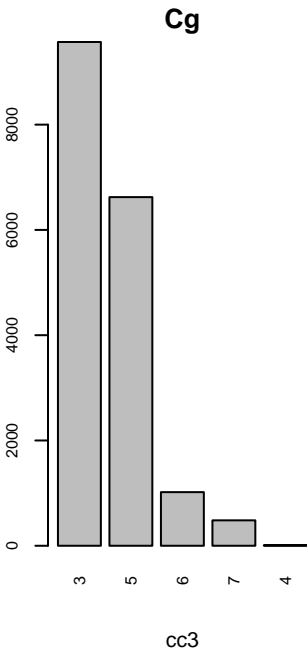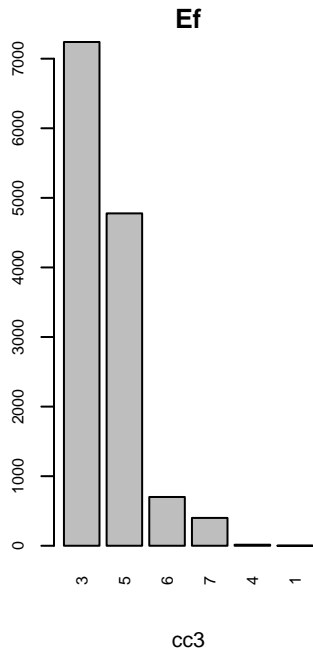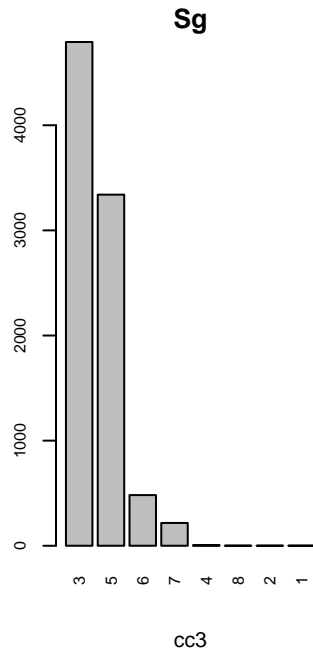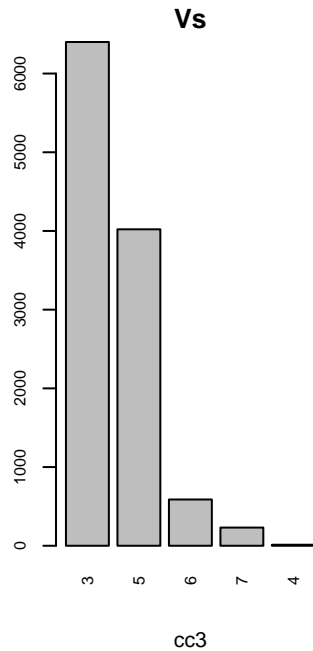

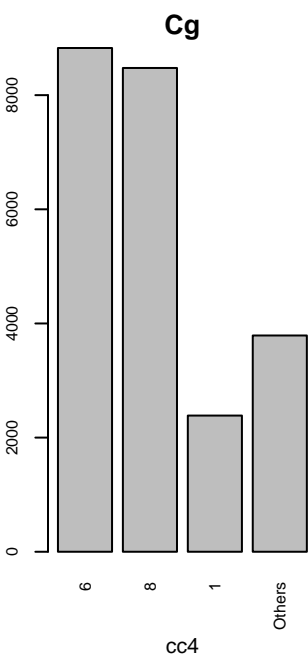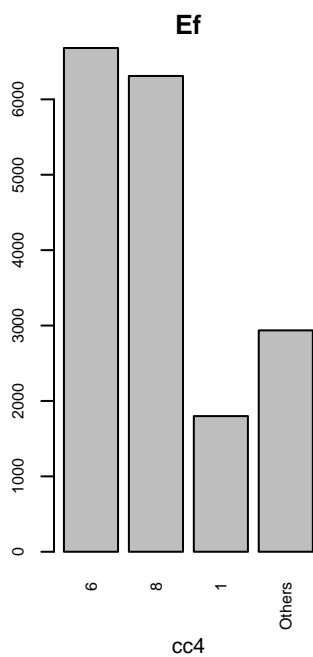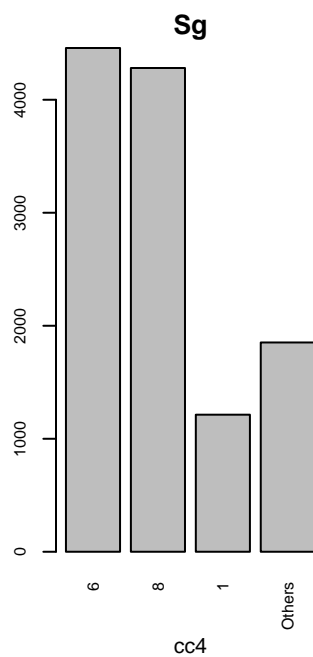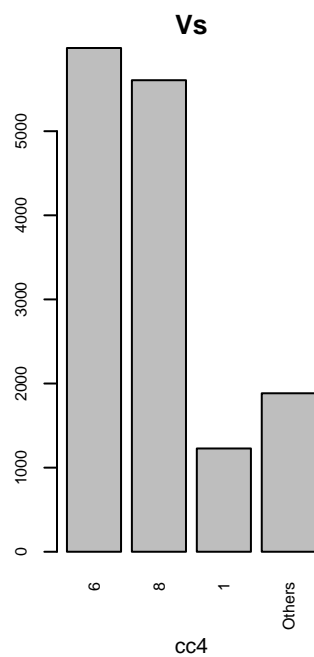

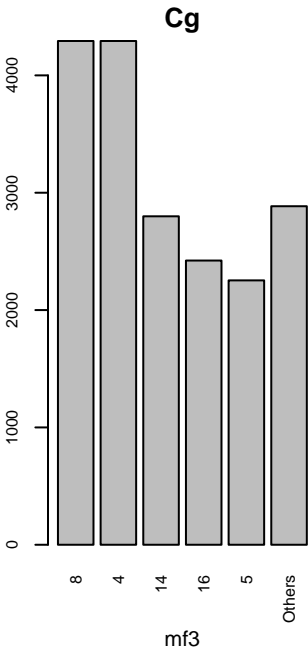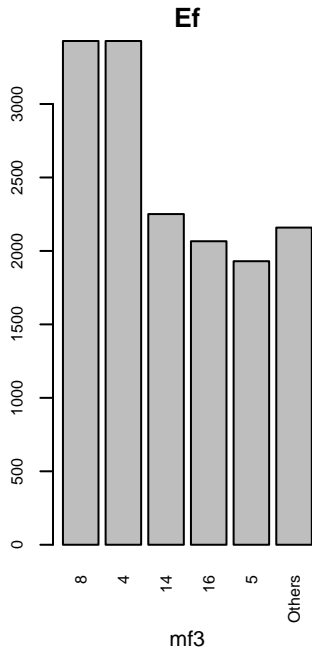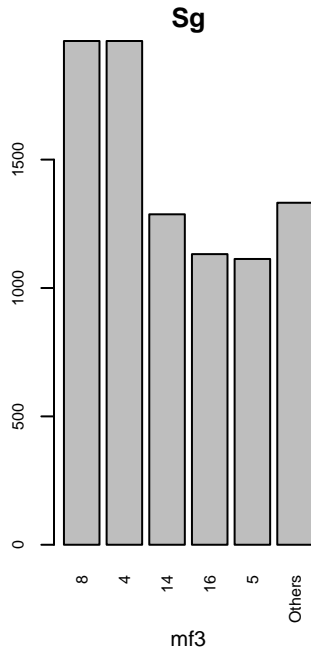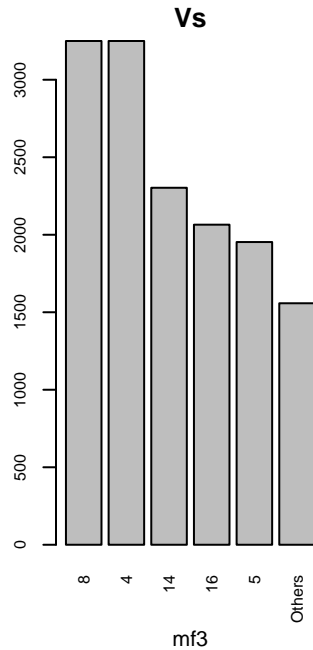

**Cg**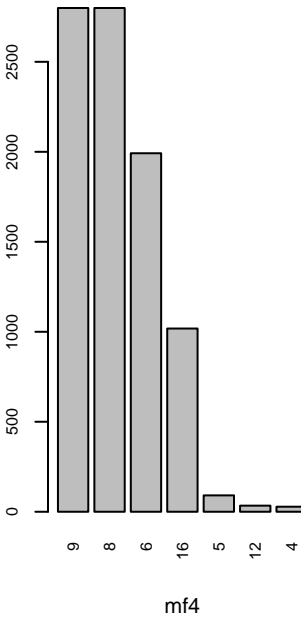**Ef**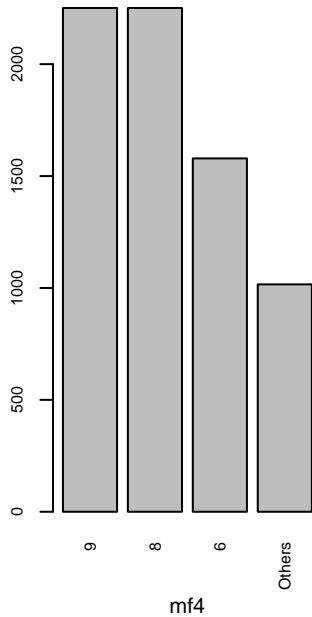**Sg**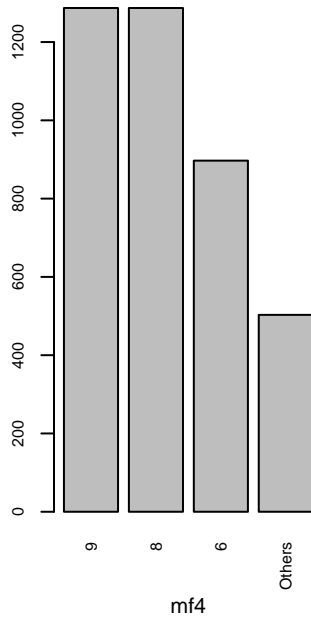**Vs**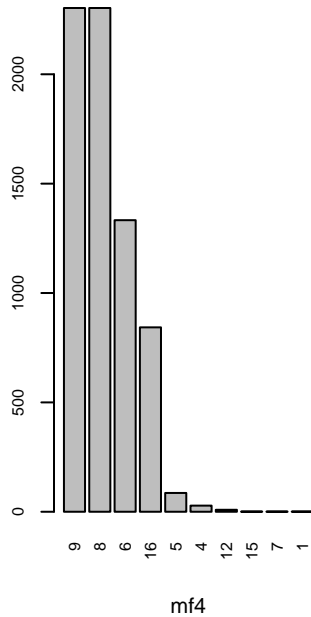

**Cg**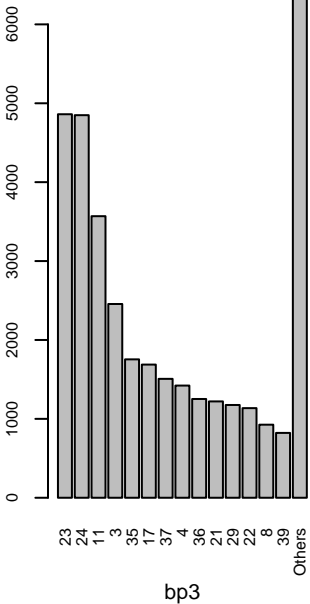**Ef**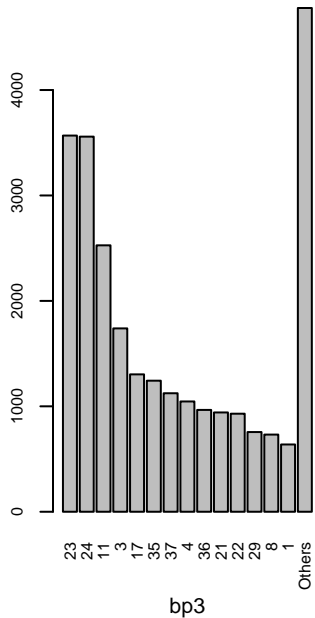**Sg**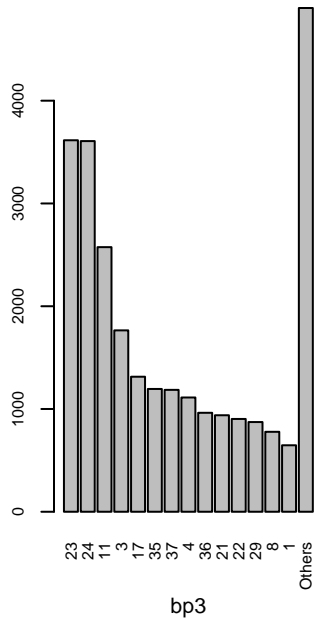**Vs**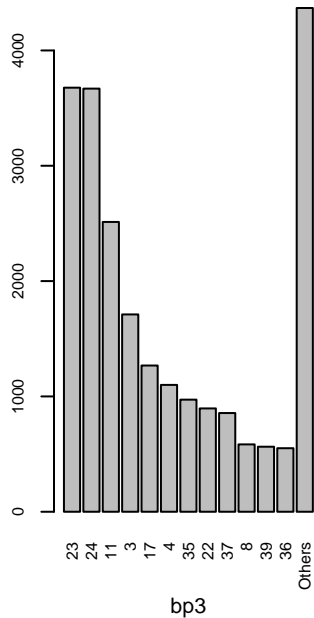

**Cg**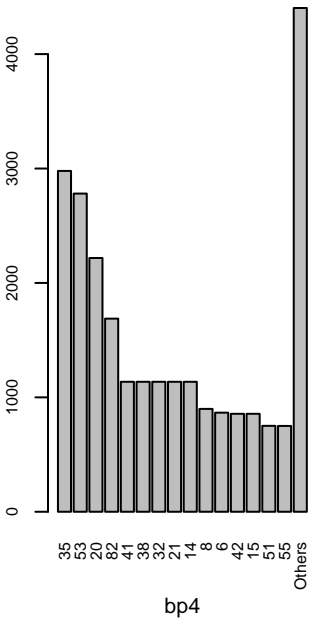**Ef**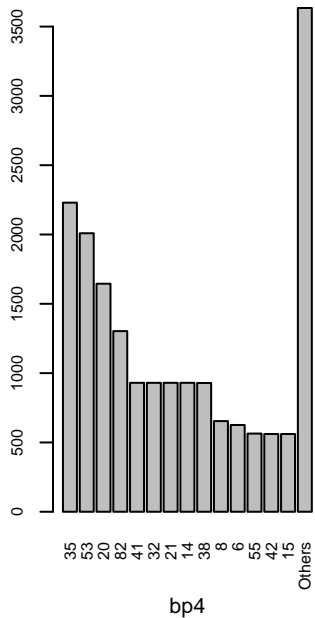**Sg**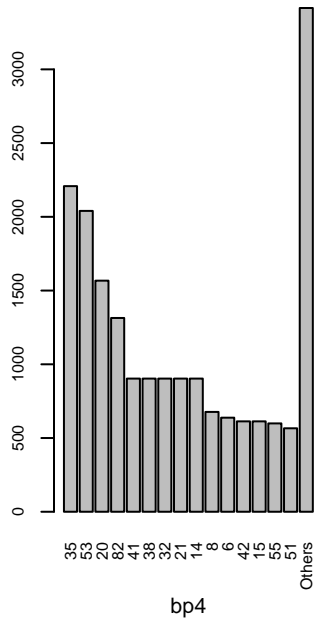**Vs**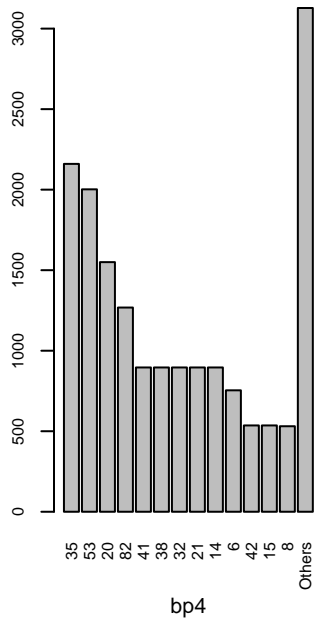

Supplement: Supplementary file 11 — Additional file 11: Figure S5: Number of contigs (y axis) per GO term per species at levels 3 and 4 for cellular components, molecular functions and biological processes. For analyses where ten or fewer GO terms appear, bars are shown for all GO terms. For analyses where more than ten terms appear, only the bars for the smallest set of terms summing up to 80% of the contigs are shown. Pane 1: Cellular components, level 3; pane 2: Cellular components, level 4; pane 2: Molecular functions, level 3; pane 4: Molecular functions, level 4; pane 5: Biological processes, level 3; pane 6: Biological processes, level 4. Species identity: Cg: Carapa guianensis; Ef: Eperua falcata; Sg: Symphonia globulifera; Vs: Virola surinamensis. Identity codes for GO terms: see Additional file 12. (PDF 23 KB) [file 12864_2014_7044_MOESM11_ESM.pdf]

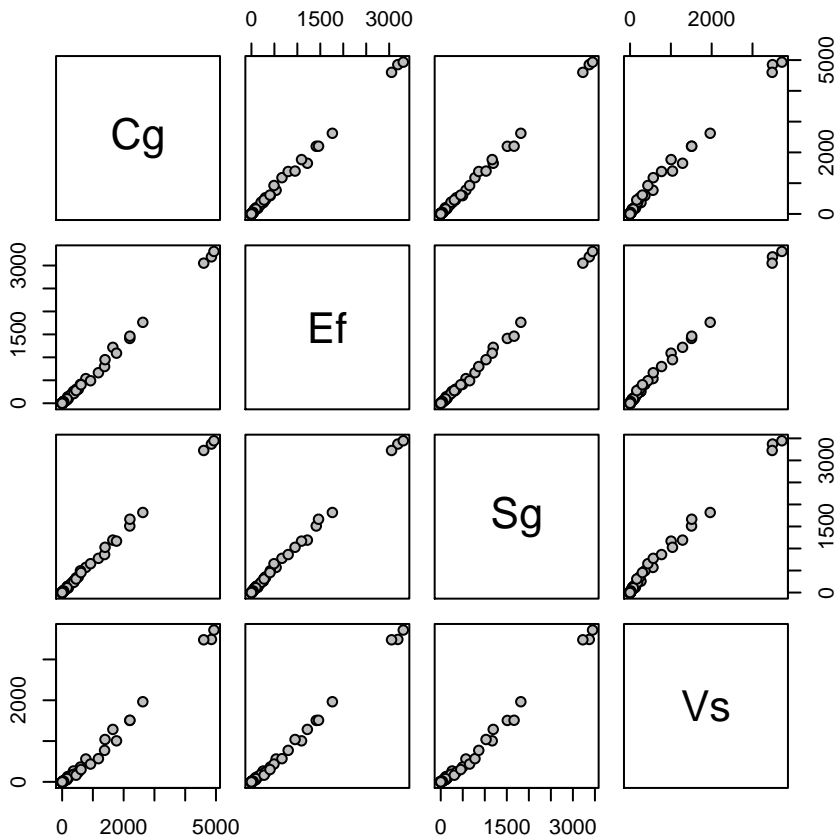

Supplement: Supplementary file 14 — Additional file 14: Figure S6: Species-species pairwise plot of the number of contigs belonging to each biological process (GO terms level 3). (PDF 10 KB) [file 12864_2014_7044_MOESM14_ESM.pdf]
