# Supplementary material for: High-throughput transcriptome sequencing and preliminary functional analysis in four Neotropical tree species
Source: BMC Genomics. 2014 Mar 27;15(1):238. doi: 10.1186/1471-2164-15-238 (PMC3986928; doi:10.1186/1471-2164-15-238)
Supplement: Supplementary file 12 — Additional file 12: Complementary caption to Figure S5. Identity codes for GO terms in Additional file 11: Figure S5. (DOCX 13 KB) [file 12864_2014_7044_MOESM12_ESM.docx]

Cellular components level 3: 1: apoplast; 2: cell-cell junction; 3: cell part; 4: extracellular region part; 5: membrane-bounded organelle; 6: non-membrane-bounded organelle; 7: organelle part; 8: protein complex.

Cellular components level 4: 1; cell periphery; 2; endomembrane system; 3; envelope; 4; external encapsulating structure; 5; extracellular space; 6; intracellular; 7; intracellular organelle part; 8; intracellular part; 9; organelle envelope; 10; organelle lumen; 11; organelle membrane; 12; organelle subcompartment; 13; plasma membrane; 14; plasmodesma; 15; proteinaceous extracellular matrix.

Molecular functions Level 3: 1: carbohydrate binding; 2: chromatin binding; 3: enzyme activator activity; 4: heterocyclic compound binding; 5: hydrolase activity; 6: ion binding; 7: lipid binding; 8: organic cyclic compound binding; 9: oxidoreductase activity; 10: oxygen binding; 11: protein binding; 12: sequence-specific DNA binding transcription factor activity; 13: signal transducer activity; 14: small molecule binding; 15: substrate-specific transporter activity; 16: transferase activity; 17: transmembrane transporter activity

Molecular functions Level 4: 1: anion binding; 2: cation binding; 3: cytoskeletal protein binding; 4: hydrolase activity, acting on acid anhydrides; 5: hydrolase activity, acting on ester bonds; 6: nucleic acid binding; 7: nucleoside binding; 8: nucleoside phosphate binding; 9: nucleotide binding; 10: oxidoreductase activity, acting on CH-OH group of donors; 11: oxidoreductase activity, acting on the CH-CH group of donors; 12: receptor binding; 13: ribulose-1,5-bisphosphate carboxylase/oxygenase activator activity; 14: substrate-specific transmembrane transporter activity; 15: transferase activity, transferring glycosyl groups; 16: transferase activity, transferring phosphorus-containing groups.

Biological processes Level 3: 1: anatomical structure development; 2: behavior; 3: biosynthetic process; 4: catabolic process; 5: cell growth; 6: cell wall organization or biogenesis; 7: cellular component biogenesis; 8: cellular component organization; 9: cellular developmental process; 10: cellular localization; 11: cellular metabolic process; 12: cellular process involved in reproduction; 13: cellular response to stimulus; 14: death; 15: detection of stimulus; 16: developmental growth; 17: establishment of localization; 18: hormone metabolic process; 19: macromolecule localization; 20: multi-multicellular organism process; 21: multicellular organismal development; 22: nitrogen compound metabolic process; 23: organic substance metabolic process; 24: primary metabolic process; 25: regulation of biological process; 26: regulation of biological quality; 27: regulation of molecular function; 28: reproductive process; 29: response to abiotic stimulus; 30: response to biotic stimulus; 31: response to chemical stimulus; 32: response to endogenous stimulus; 33: response to external stimulus; 34: response to other organism; 35: response to stress; 36: single-multicellular organism process; 37: single-organism cellular process; 38: single-organism developmental process; 39: single-organism metabolic process; 40: single organism signalling.

Biological processes Level 4: 1: abscission; 2: aging; 3: anatomical structure morphogenesis; 4: auxin metabolic process; 5: carbohydrate derivative metabolic process; 6: carbohydrate metabolic process; 7: cell-cell signaling; 8: cell communication; 9: cell cycle; 10: cell death; 11: cell differentiation; 12: cell division; 13: cell wall biogenesis; 14: cellular aromatic compound metabolic process; 15: cellular biosynthetic process; 16: cellular catabolic process; 17: cellular component assembly; 18: cellular homeostasis; 19: cellular macromolecule localization; 20: cellular macromolecule metabolic process; 21: cellular nitrogen compound metabolic process; 22: defense response; 23: detection of chemical stimulus; 24: developmental growth involved in morphogenesis; 25: developmental maturation; 26: developmental process involved in reproduction; 27: embryo development; 28: establishment of localization in cell; 29: fruit ripening; 30: generation of precursor metabolites and energy; 31: glycosyl compound metabolic process; 32: heterocycle metabolic process; 33: homeostatic process; 34: lipid metabolic process; 35: macromolecule metabolic process; 36: microtubule-based process; 37: multi-organism reproductive process; 38: nucleobase-containing compound metabolic process; 39: organelle organization; 40: organic acid metabolic process; 41: organic cyclic compound metabolic process; 42: organic substance biosynthetic process; 43: organic substance catabolic process; 44: organonitrogen compound metabolic process; 45: organophosphate metabolic process; 46: oxidation-reduction process; 47: phosphorus metabolic process; 48: photosynthesis; 49: plant-type cell wall organization or biogenesis; 50: positive regulation of molecular function; 51: post-embryonic development; 52: protein localization; 53: protein metabolic process; 54: regulation of catalytic activity; 55: regulation of cellular process; 56: regulation of developmental process; 57: regulation of hormone levels; 58: regulation of localization; 59: regulation of metabolic process; 60: regulation of multicellular organismal process; 61: regulation of response to stimulus; 62: regulation of signaling; 63: response to bacterium; 64: response to cold; 65: response to extracellular stimulus; 66: response to hormone stimulus; 67: response to inorganic substance; 68: response to karrikin; 69: response to nematode; 70: response to nitrogen compound; 71: response to organic substance; 72: response to organonitrogen compound; 73: response to oxygen-containing compound; 74: response to radiation; 75: response to temperature stimulus; 76: response to water deprivation; 77: response to water stimulus; 78: secondary metabolic process; 79: small molecule metabolic process; 80: system development; 81: tissue development; 82: transport; 83: tropism.
