# Supplementary material for: High-throughput transcriptome sequencing and preliminary functional analysis in four Neotropical tree species
Source: BMC Genomics. 2014 Mar 27;15(1):238. doi: 10.1186/1471-2164-15-238 (PMC3986928; doi:10.1186/1471-2164-15-238)
Supplement: Supplementary file 15 — Additional file 15: Complementary Caption to Figure 4 . Detailed names of the biological processes shown in Figure 4. (DOCX 12 KB) [file 12864_2014_7044_MOESM15_ESM.docx]

**Detailed names of the biological processes shown in Fugure 4.** (A) C. guianensis: Leaves (nitrogen cycle metabolic process, carbon fixation, photosynthesis, response to temperature stimulus, response to cold, response to oxidative stress, carbohydrate metabolic process, generation of precursor metabolites and energy, response to radiation, oxidation reduction process); Stems (ribonucleoprotein complex biogenesis, microtubule based process, cellular component movement, response to karrikin, negative regulation of molecular function, organic substance catabolic process, cellular ketone metabolic process, regulation of metabolic process, secondary metabolic process), Roots (aging, ribonucleoprotein complex biogenesis, negative regulation of molecular function, defense response, transmembrane transport, response to oxygen containing compound, response to organic substance); (B) E. falcata: Leaves (carbon fixation, photosynthesis, generation of precursor metabolites and energy, oxidation reduction process, carbohydrate metabolic process, organic substance biosynthetic process, cellular biosynthetic process), Stems (actin filament based process, regulation of immune system process, regulation of innate immune response , activation of innate immune response, positive regulation of molecular function, reactive oxygen species metabolic process, olefin metabolic process, cellular alkene metabolic process, one carbon metabolic process, cellular component movement, photosynthesis, ribonucleoprotein complex biogenesis, sulfur compound metabolic process, single organism biosynthetic process, secondary metabolic process, carbohydrate metabolic process, carbohydrate derivative metabolic process, organic substance biosynthetic process, cellular biosynthetic process), Roots (actin filament based process, regulation of immune system process, activation of innate immune response, positive regulation of molecular function, reactive nitrogen species metabolic process, nitrogen cycle metabolic process, reactive oxygen species metabolic process, cellular alkene metabolic process, cell death, one carbon metabolic process, cellular membrane organization, response to heat, response to oxidative stress, response to inorganic substance, cellular response to chemical stimulus, oxidation reduction process, single organism biosynthetic process, secondary metabolic process, cellular ketone metabolic process, small molecule metabolic process, glycosyl compound metabolic process, organic substance biosynthetic process, cellular biosynthetic process, response to osmotic stress, cellular catabolic process, cellular response to stress); (C) S. globulifera: Leaves(root morphogenesis, carbon fixation, photosynthesis, nitrogen cycle metabolic process, generation of precursor metabolites and energy, oxidation reduction process, response to radiation), Stems (fruit ripening, reactive oxygen species metabolic process, photosynthesis, response to oxidative stress, response to heat, secondary metabolic process, generation of precursor metabolites and energy, defense response); (D) V. surinamensis: Leaves (photosynthesis, generation of precursor metabolites and energy, carbon fixation, response to water deprivation, response to cold), Stems (activation of innate immune response, one carbon metabolic process, photosynthesis, ribonucleoprotein complex biogenesis,translational initiation, generation of precursor metabolites and energy, response to other organism, defense response, cellular response to chemical stimulus, response to oxidative stress, reactive oxygen species metabolic process, secondary metabolic process, organic substance catabolic process, cellular catabolic process, single organism biosynthetic process, response to osmotic stress, response to water stimulus, response to water deprivation, response to oxygen containing compound, organic substance biosynthetic process, cellular biosynthetic process, response to inorganic substance, response to heat, response to temperature stimulus, organic substance transport), Roots (activation of innate immune response, response to oxidative stress, reactive oxygen species metabolic process, secondary metabolic process, cellular catabolic process, single organism biosynthetic process ,response to osmotic stress, response to water stimulus, response to water deprivation, response to oxygen containing compound, response to organic substance, transmembrane transport, positive regulation of biological process, response to inorganic substance); (Note: sequencing from S. globulifera roots failed).
